# Development of a Non-Spherical Polymeric Particles Calibration Procedure for Numerical Simulations Based on the Discrete Element Method

**DOI:** 10.3390/polym17202748

**Published:** 2025-10-14

**Authors:** Joshua García-Montagut, Rubén Paz, Mario Monzón

**Affiliations:** Mechanical Engineering Department, Edificio de Ingenierías, Campus de Tafira Baja, Universidad de Las Palmas de Gran Canaria, 35017 Las Palmas, Spain; ruben.paz@ulpgc.es

**Keywords:** discrete element method, polymeric powder calibration, material calibration, genetic algorithms

## Abstract

The manufacturing industry, in general, and the plastic industry, in particular, have been developing new materials and process methods that need a correct study and optimization. Nowadays, the main approach to optimize these processes is using numerical methods and, in the case of particulate materials, the Discrete Elements Method to estimate the particles interactions. But those mathematical models use some parameters that depend on the material and must be calibrated, thus requiring an important computational and experimental cost. In this study, we integrate different speed-up procedures and present a general calibration method of Low-Density Polyethylene particles, to obtain the calibrated solid density and Poisson’s ratio of the material, the restitution, static and rolling friction factors in the particle-to-particle and particle-to-wall interactions, and the contact model variables (damping factor, stiffness factor, and energy density). For this calibration, four different tests were carried out, both experimentally and with simulations, obtaining the bulk density, the repose and shear angles, and the dropped powder. All these response variables were compared between simulations and experimental tests, and using genetic algorithms, the input parameters (design variables) were calibrated after 85 iterations, obtaining a Mean Absolute Percentage Error of the response variables lower than 2% compared to the experimental results.

## 1. Introduction

There are several polymer processes where the feedstock material can be used in powder or particle form. This is the case with conventional processes such as rotational molding, extrusion, compression molding, or sintering under additive manufacturing such as the technology of Powder Bed Fusion (PBF) [1].

Due to the complexity of all those manufacturing methods and the costs associated, it is necessary to study the behavior of the powder during the process in the machine, mainly before the melting stage. For this, nowadays the use of numerical methods is generalized to predict the process conditions and final quality of the part. Additionally, computational power has increased and allows us to know more and more precisely the material behavior inside these simulated processes. Specially, the use of Discrete Elements Method (DEM) allows us to understand how the particles interact with other particles and with the boundaries.

DEM is based on the use of mathematical models that represent the particle contact, having different models depending on the characteristics of the particles being studied; and the correct selection has a great impact on the final simulations [2]. Those mathematical models are based on the use of certain parameters that depend on the model chosen, the boundary material, and the particle material in each case. The process of obtaining these parameters is called the calibration process and is widely studied in the bibliography. There are several calibration methods, but all of them focus on the speeding-up of the calibration.

One speed-up method is scaling up the particles. The DEM is applied to each particle for each iteration, and this means a direct relationship between the number of particles and the calculation time. If we are able to scale-up the particles, there will be fewer particles for the same volume of powder. Thakur et al. [3] and Roessler et al. [4] concluded that it is possible to scale up the system or the particle when the dimensions of the system are not comparable to those of the particles. In this context, Remy et al. [5] observed a minimum relation needed between the system space and the particle diameter of 31.5 times to guarantee the elimination of the wall friction effect.

When the particles are scaled, the generated forces change too; those stress relations are called scale law and have been studied by some authors such as Janda and Ooi [6]. Following these laws, Coetzee [7] established the scaling factor limit up to 1.3–2.5 for particle discharge operations and 4 times for dynamic operations. Lommen et al. [8] calculated a limit scaling factor of 3–5 times, but it was limited by the system environment. However, unlike the limits shown before, Yeom and Choi [9] validated a dynamic study with a particle scaling factor of 100 times the particle diameter.

With the same objective of reducing the number of particles, Ahmadi et al. [10] studied the option of truncating the particle distribution from a diameter and unifying the diameter of the smaller particles in different percentages (scalping method). This process increases the minimum diameter of the distribution, and as the time step of the simulation is proportional to the minimum diameter of powder, it reduces the number of steps and the computational time by up to 90%.

The process for obtaining the correct combination of parameters during the calibration usually is by trial and error [11,12]; this means that it is necessary to do a large number of tests to find it. Trying to reduce the number of iterations is another way to speed up the calibration process. Focusing on this reduction, there are three main lines of study: advanced design of experiments (DoE), artificial neuronal networks, and evolutionary algorithms.

El-Kassem et al. [13,14] showed in two different studies a methodology based on the multivariate regression analysis (MVRA), establishing which parameters affect each calibration test type. Hanley et al. [12] studied the significance of the different variables using a two-dimensional ANOVA study in the first step and a three-dimensional ANOVA study for the most significant variables from the previous step. A DoE based on the definitive screening design (DSD) method was chosen by Bhalode and Ierapetritou [15] to analyze the impact of each calibration factor, obtaining a predicting equation for each test and material.

Orefice [16] introduced a way to reduce the number of iterations using artificial neural networks and tests chosen in such a way that the predominant variables overlapped at least two by two. Something similar was studied by [17]. Zhou et al. used a Latin Hypercube sampling for the initial sampling points.

Finally, using a genetic algorithm (NSGA-II optimization algorithm), Mohajeri et al. [18] proved the possibility of using this kind of algorithm in any contact model or configuration setup. With the same algorithm, Do et al. [19] applied a multi-objective optimization, fitting the model to the real test and reducing the computational time. Despite this, Rackl and Hanley [20] needed almost 51 iterations in the best case using Latin Hypercube sampling and the Kriging interpolation method.

All the studies shown before used different tools for speeding up the calibration individually, but there are no studies about the possibility of decreasing the calibration time using most of these techniques in combination.

In this study, a new calibration process is presented using the scaling-up and scalping particles methods for reducing the computational time. To reduce the number of iterations, an initial population generated by a modified Latin Hypercube sampling and a subsequent genetic algorithm application based on the Kriging interpolation method were chosen.

## 2. Materials and Methods

**Figure 1:**
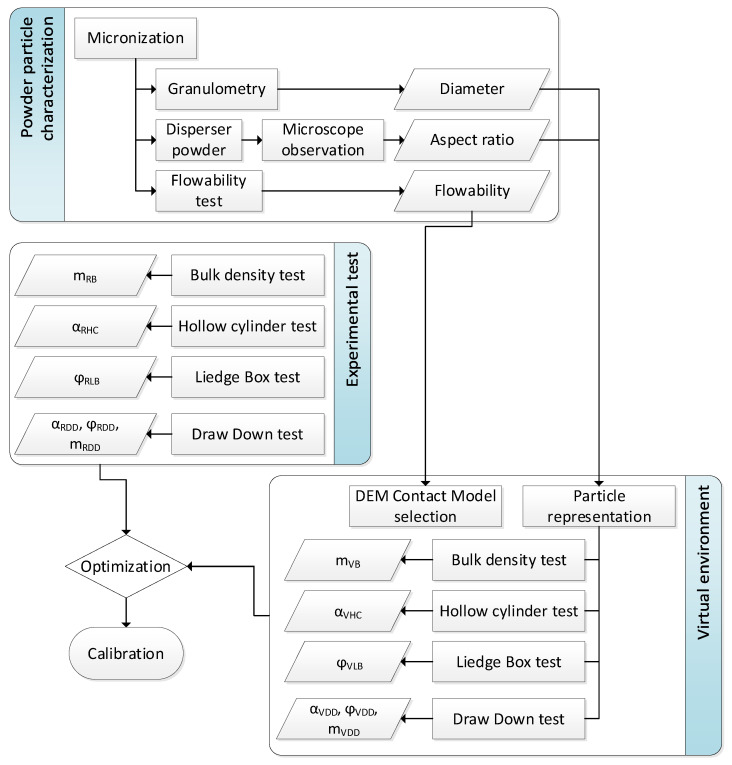
Experiment flow chart.

This method is divided into a first part of experimentation, where the particles are characterized and the tests are carried out; after this, in the second stage, the particles are represented in the DEM simulation setup with a representation of the experimental test, and the final stage is the optimization using the results of the experiments and the simulation. All the process is summarized in Figure 1.

### 2.1. Materials

For the realization of this experiment, Low-Density Polyethylene LDPE 1200 MN 18 C, by Total (UUEE), Valencia, Spain, has been used. It is a thermoplastic polymer with a density of 0.918 g/cm^3^.

LDPE is a material that presents very good characteristics both from the point of view of its mechanical characteristics and its recyclability. The report about the plastic industry situation from the Plastic Europe Marketing Report Group (PEMRG) places LDPE as the second most used plastic, only behind polypropylene [21].

### 2.2. Experimentation

#### 2.2.1. Powder Preparation and Shape Characterization

The material is provided in pellet format with 3 mm of approximate diameter. Since the material is needed in powder format, these pellets were micronized by mechanical methods with a centrifugal mill Retsch ZM 200 (Verder, Germany). This machine has 1300 W of power, and the configuration chosen was 10,000 rpm with a 500 µm sieve.

Since the powder particles obtained did not have a uniform size, a Mastersizer 2000 (Malvern Panalytical, Spain) was used to measure the particle size (laser diffraction technique).

Due to the intrinsic characteristics of the micronization process, the particles obtained had a random shape. Because the aspect ratio (Equation (1)) is needed for representing the particle in the simulation, an Olympus BX51 Fluorescence Microscope (Plympus, Japan) was used to observe the particle shapes and be able to calculate the aspect ratio, taking pictures from the observation and measuring them.

(1) $$AR=\frac{Particle\;length}{Particle\;width}$$

**Figure 2:**
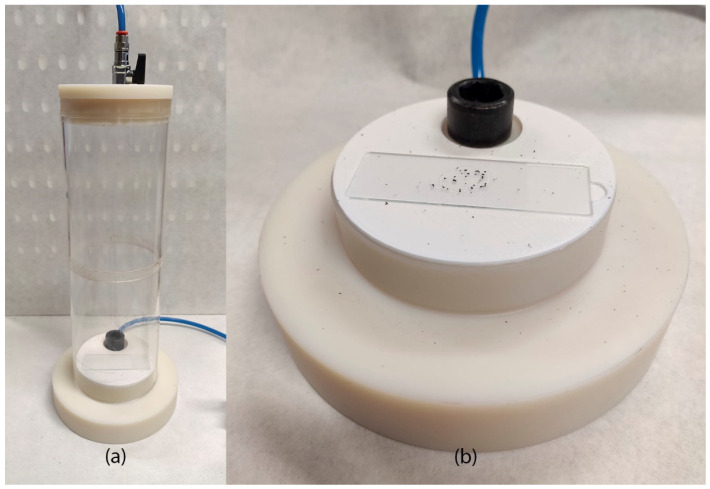
Vacuum powder disperser (**a**) and slide with dispersed powder (**b**).

In order to do this observation, a vacuum powder disperser (Figure 2a) was developed and used to prepare slides of isolated particles without stacking. This device generates a vacuum chamber; when a valve is open, the powder is projected from the bottom to the top part of the chamber. Once the atmospheric pressure is re-established, the powder drops to the bottom again in an isolated form on the slide with a sticky surface, enabling the shape characterization (Figure 2b).

After observation, the shape and the aspect ratio of each group of particles were obtained (almost 5 measurements of each group). The resulting shape and aspect ratio of each group are depicted in Section 3.2.1. This information was used as input for the simulations carried out, which are explained in Section 2.3.

#### 2.2.2. Determination of the Bulk Density, Tap Density, and Flowability

When a determined amount of powder is placed on a flat surface without receiving external forces (except gravity), the powder tends to agglomerate itself, leaving little gaps between particles. The bulk density ($\rho_{B}$) is the relationship between the volume ($V_{B}$) and the weight of this powder (without having been compacted) (Equation (2)).

(2) $$\rho_{B}=\frac{Sample\;weight}{Volume\;without\;compacting}$$

If this powder without compacting is subjected to different stress, the powder tends to fill the air gaps, reducing the volume and increasing the density. This relationship between the weight with respect to the new volume ($V_{T}$) is called tap density ($\rho_{T}$), shown in Equation (3).

(3) $$\rho_{T}=\frac{Sample\;weight}{Compacted\;volume}$$

**Figure 3:**
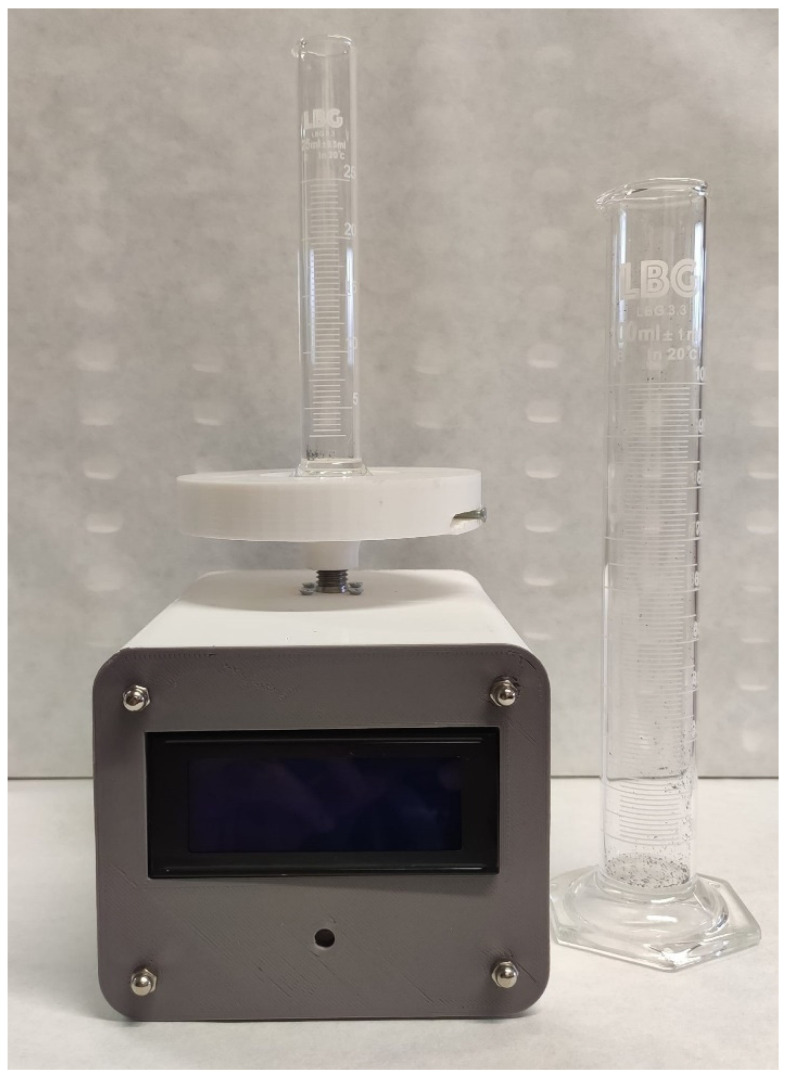
Tap density test machine.

To guarantee a proper comparison environment between particles, the compaction stress was applied using a machine developed for this purpose (Figure 3), following the standard ISO 3953:2011 [22]. This machine generates a known and controlled axial pulsated movement according to the standard. This axial movement allows the compaction of the powder.

This standard establishes the use of a cylinder with a 100 cm^3^ volume capacity that is filled with 50 g of powder; this cylinder is stirred at a controlled frequency (normally 300 pulses per minute). While the standard focuses on metal powders, this study uses LDPE. Therefore, for the same weight, we had too much volume, and the 100 cm^3^ cylinder had insufficient capacity. For this reason, 20 g of plastic powder were used. The volume occupied by the powder, before and after the stirring was applied, was determined, thus obtaining the bulk ($\rho_{B}$) and tap ($\rho_{T}$) densities.

With the previous densities, the Carr’s Index (*CI*%, Equation (4)) and the Hausner ratio ((*H*), Equation (5)) can be calculated.

(4) $$CI\%=100\cdot \frac{\rho_{T}-\rho_{B}}{\rho_{T}}$$

(5) $$H=\frac{\rho_{T}}{\rho_{B}}$$

Both are indexes that quantify the flowability of dry powder according to Table 1.

#### 2.2.3. Experimental Reference Tests

Several experimental tests were selected and carried out to use the output results as reference values for the calibration process. Among all the available tests, according to the literature, the Static Angle of Repose (α) in the Hollow Cylinder test is one of the most commonly used tests to obtain the interaction coefficients between particles [24]. However, Katterfeld et al. [25,26] highlighted the ambiguity of the results when only one test is used. For this reason, we carried out an additional Liedge box test that provides the static friction angle (ϕ) [27,28]. In addition, a bulk density test was conducted because this measurement (bulk density) could change during the calibration. Finally, a drawdown test was also carried out to obtain other repose and friction angles, as well as the discharged mass. The following subsections explain all these experimental tests.

##### Bulk Density Test

**Figure 4:**
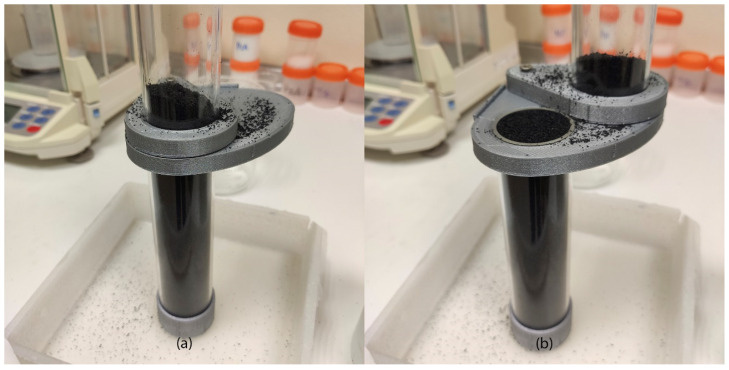
Alternative bulk density measuring device in initial position (**a**) and device sectioned with the selected amount of powder for mass measuring for determining the bulk density (**b**).

In this research, experimental and simulated tests are compared to conduct the calibration. In the case of the bulk density, this data was measured with the method explained in Section 2.2.2; however, replicating that test in a simulated environment needs too much computational power to be reproduced. Therefore, a different bulk density measuring device was used for more volumetric accuracy and for easier replicability in a virtual environment. The device developed for this purpose is composed of two cylinders with a diameter of 18.05 mm and a total height of 301.78 mm (one on top of the other) (Figure 4a). These cylinders are held in alignment by a structure that allows them to be pivoted in the horizontal plane without applying axial stress to the powder, avoiding the compaction, leaving a cylinder of 158.65 mm high full of bulk powder (Figure 4b). In this test, 200 mL of powder was used, and the mass of powder obtained in the cylinder ($m_{RB}$) was measured.

##### Hollow Cylinder Test

**Figure 5:**
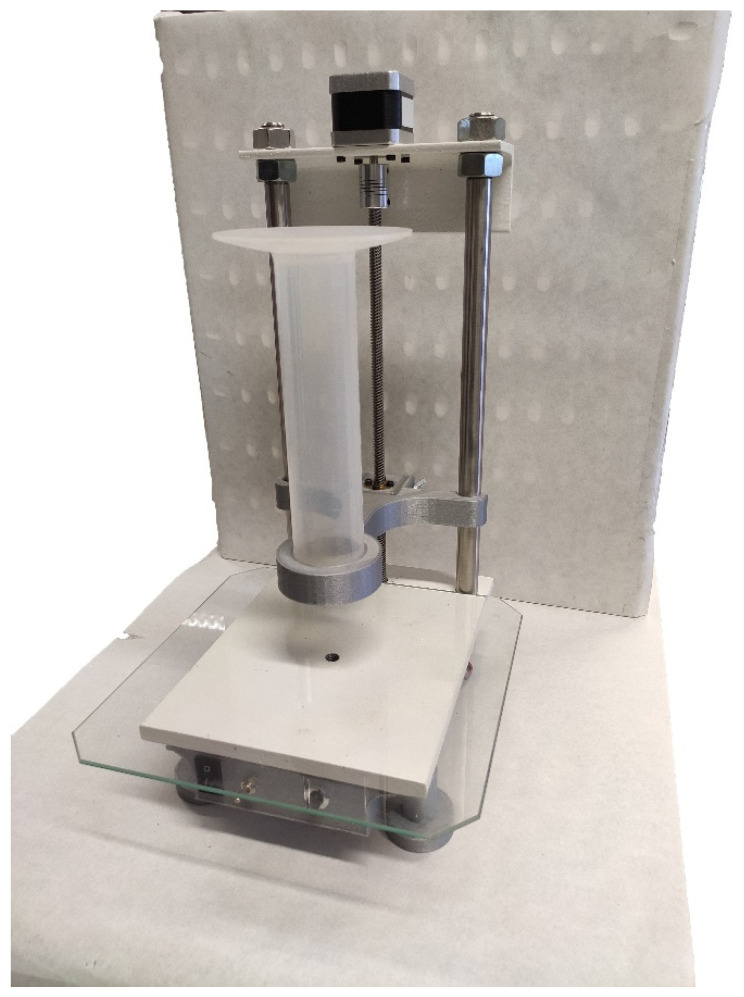
Hollow cylinder machine.

The second reference test is the hollow cylinder method. The machine used in this case (Figure 5) has three different parts: structure, hollow cylinder, and dropping base.

The cylinder in this machine has an inner diameter of 38 mm.

**Figure 6:**
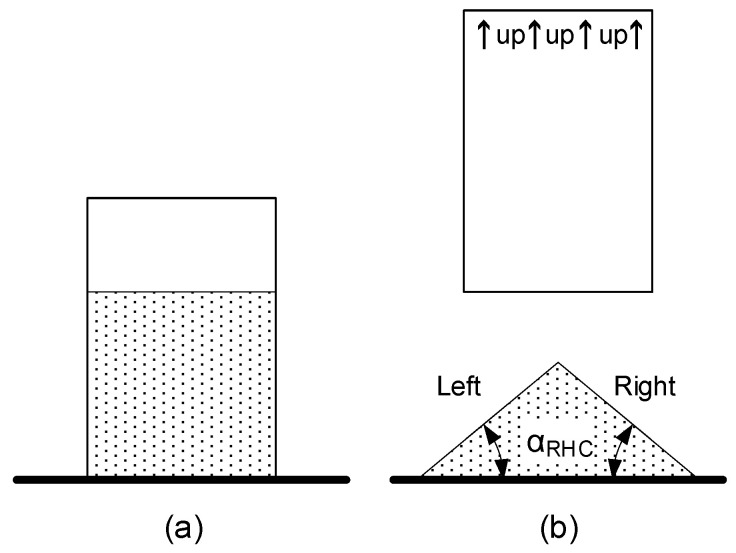
Hollow cylinder test principle starting test (**a**) and finishing test with the measured angles (**b**).

This method consists of filling a cylinder with a determined volume of bulk powder, raising this cylinder with a controlled speed, and seeing the angle of repose (α_RHC_) of the powder pile (Figure 6). The angle of repose is measured on both sides (left and right) of the pile, and the average is calculated.

In this test, the results are affected by many parameters (elevation speed, cylinder shape, base, and cylinder materials, etc.), but in previous studies [29,30], the authors indicated that to guarantee the tapered powder pile shape, it is better to use a ratio (a) between the powder height ($H_{i}$) before the test and the cylinder radio ($R_{i}$) with a value under 3 (6).

(6) $$a=\frac{H_{i}}{R_{i}}$$

Consequently, we decided to use a=2, and, with our hollow cylinder machine ($R_{i}$ = 19 mm), a 38 mm powder column was used in the test, equivalent to 40 cm^3^.

In addition, Li et al. [31,32] and Cao et al. indicated that, in our case, it is better to maintain a slow elevation speed, so we selected 0.01 m/s of elevation speed.

The angle of repose of this test (α_RHC_) was measured by image processing (method explained in Section 2.4).

##### Liedge Box Test

**Figure 7:**
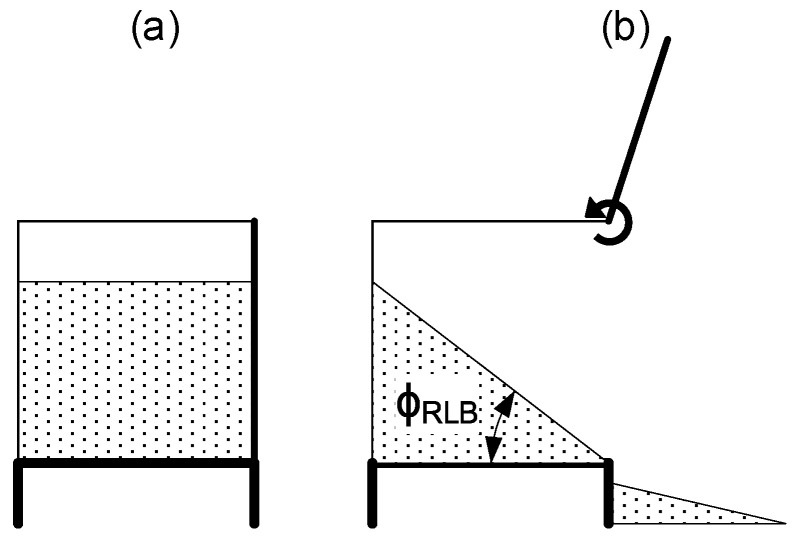
Ledge box test principle starting test (**a**) and finishing test with the measured angles (**b**).

The third test is the Liedge box test. In this test there is a dimensional limitation highlighted by Derakhshani et al. [33], who established a minimum distance between walls inside the box must be 32 times the particle diameter. In this test we used an acrylic box with 75 × 100 × 16 mm inner dimensions filled with bulk powder. The right wall can be rapidly opened by hand, eliminating the restriction and letting the powder flow freely in that direction (Figure 7). The amount of powder that remains in the box allows us to measure the internal friction angle (ϕ_RLB_) by image processing (method explained in Section 2.4).

For this test, a powder volume of 120 cm^3^ was used.

##### Draw Down Test

The last test is the drawdown test. The device is an acrylic box divided into two parts (one on the top and another on the bottom) with a centered gate between them. Coetzee [34] established the powder heap at a width of 10 to 24 times the particle diameter and the gate width at 6 to 13 times the particle diameter. Additionally, several publications [15,34,35] set a relation between the width and the length of a powder heap of 5 times.

**Figure 8:**
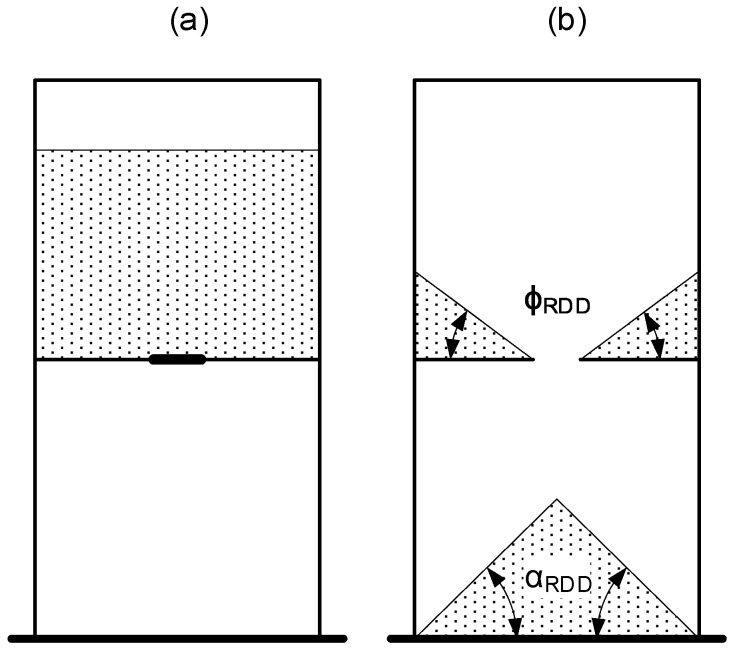
Drawdown test principle starting test (**a**), finishing test with the measured angles (**b**).

Finally, the total inner dimension box was 125 × 60 × 12 mm, and 6.5 mm of gate width. The test consists of filling the upper part with powder, waiting for one second for powder stabilization, and opening the gate (Figure 8). When the powder stopped dropping, the angle of repose in the lower part (α_RDD_) and the static friction angle on both sides of the upper part (ϕ_RDD_) were measured by image processing (method explained in Section 2.4). The weight of fallen powder (m_RDD_) was measured too with a scale.

For this test, a powder volume of 21.9 cm^3^ was used.

### 2.3. Virtual Environment

This section explains all the models and setups carried out to simulate and compare the previous experimental tests.

#### 2.3.1. Contact Model DEM Parameters

**Figure 9:**
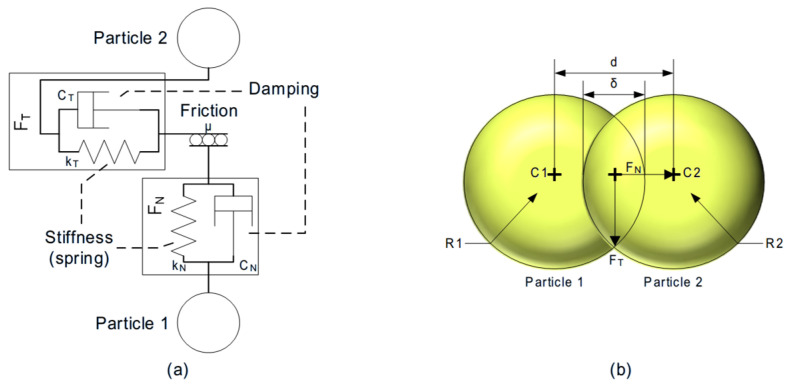
Schema of interaction representation between particles (**a**) and representation of the overlap in the contact point and their principal measures (**b**).

Polymeric particles are assumed to have some grade of elasticity. This entails that when the particles interact, some deformation occurs; in the case of particles interacting between them, the distance between their centers is reduced due to an overlap considered between both particles (Figure 9b). In Figure 9a, the interactions in the contact point of two particles are represented using a spring and a damper in parallel connection.

**Figure 10:**
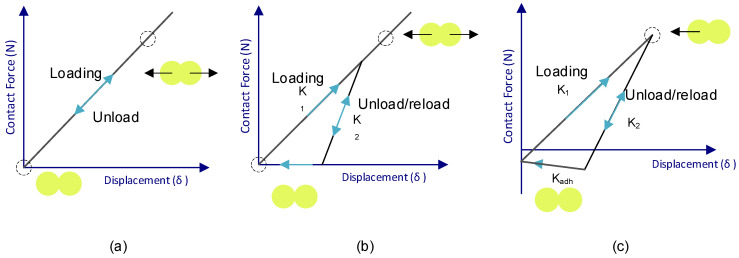
Contact force–displacement function of Hert-Mindlin (HMCM) (**a**), hysteretic spring (HSCM) (**b**), and Edinburgh elasto-plastic adhesion (EEPACM) (**c**) models.

Depending on the mathematical model used, the energy transmitted by these interactions has a minor or major representation in the movement equations. An example of that is Figure 10, where the function contact force–displacement is represented, with Figure 10a being the representation of the more basic model (Hert–Mindlin Contact Model—HMCM), Figure 10b the representation of the hysteretic spring contact model (HSCM), and Figure 10c the Edinburgh Elasto-Plastic Adhesion Contact Model (EEPACM).

Although each of these models is more accurate than the previous one, the complexity of these has an impact on the computational cost, so not always the most complete model is the most efficient one.

Following the literature, for this research the hysteretic spring contact model (HSCM) is chosen (dry powder with good flow) for the interparticle interactions [36,37] and the Hert–Mindlin model (HMCM) for the particle-wall interactions [13,38].

At the moment of contact between two particles, the force generated is divided into two components named Normal Force ($F_{N}$) and Tangential Force ($F_{T}$), as shown in Figure 9a. The definition of each force depends on the model.

The governing equations for the Hert–Mindlin (no slip) model are listed in Table 2 [39].

In the case of the HSCM, some changes are added to take into account the energy lost during contact. The governing equations are listed in Table 3 [40,41,42,43].

To represent the cohesion force between particles, the Linear Cohesion V2 Contact Model (LCCM) was integrated into the HSCM, adding the cohesive force to the total normal force ($F_{TN}$) [36,44]. There is a Linear Cohesion V1 Contact Model, but Version 2 modified the first version, changing the way the contact area is calculated. This V2 version is recommended for non-uniform particle size distributions. This model is calculated with Equation (10).

(7) $$F_{N}^{c}=\xi \cdot A_{c}$$

where ξ is the energy density (Jm^−3^) related to the cohesion (kPa) between particles. $A_{c}$ is the contact area [45], defined by Equation (8).

(8) $$A_{c}=\pi \frac{R_{i}{\cdot R}_{j}}{R_{i}{+R}_{j}}\cdot \delta_{N}$$

Finally, the total normal force is obtained by Equation (9).

(9) $$F_{TN}=F_{N}+F_{N}^{d}+F_{N}^{c}$$

#### 2.3.2. Particle Shape Design

**Figure 11:**
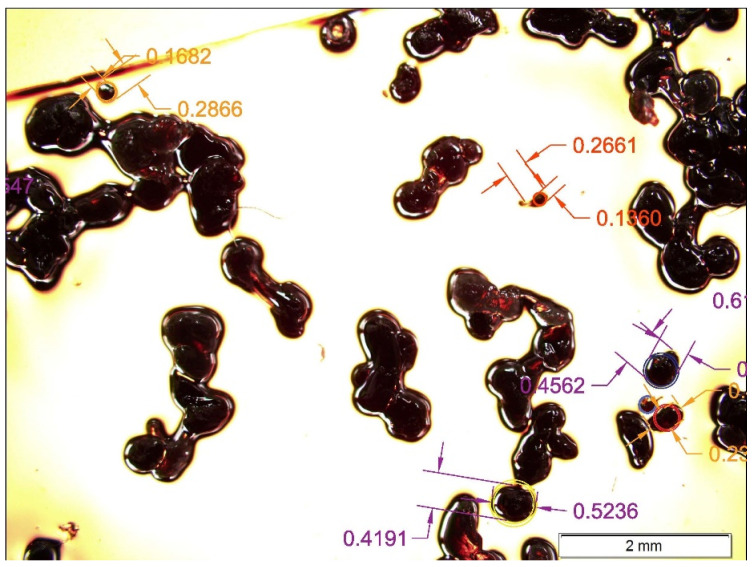
Capture of microscope visualization with aspect ratio measurements examples.

The diameter particle distribution was calculated using the Mastersizer 2000 test. Five slides were obtained using the vacuum powder disperser, and several pictures of the dispersed powder described in Section 2.2.1. were taken in the microscope, and the aspect ratio of each group of particles was calculated. An example of the measuring of particles in the picture taken during the microscope observation is shown in Figure 11. In order to ensure the correct representation of each particle group, a minimum of ten particles per slide were measured for each granulometry group.

However, it should be noted that it is not necessary to use the exact real shape the particle. Regarding particle shape complexities, Moncada et al. [46] concluded that more complex particle shapes (polyhedra) achieve a better fit during calibration (evidenced by a lower Weighted Mean Square Error, or WMSE) compared to spherical particles. However, they acknowledged that the computational cost can be up to 24 times higher for polyhedra than for simple spheres.

In contrast, Marín Pérez et al. [47] suggest that multi-sphere particles (clumps) are a computationally efficient method for modeling irregular particles when compared to using polyhedra. An approximation using 2 overlapped spheres is enough [48], maintaining the aspect ratio [49].

#### 2.3.3. Test Simulations

The experimental tests described in Section 2.2.3. were replicated in EDEM 2023 software. This subsection explains the procedure carried out.

##### Bulk Density Simulation

**Figure 12:**
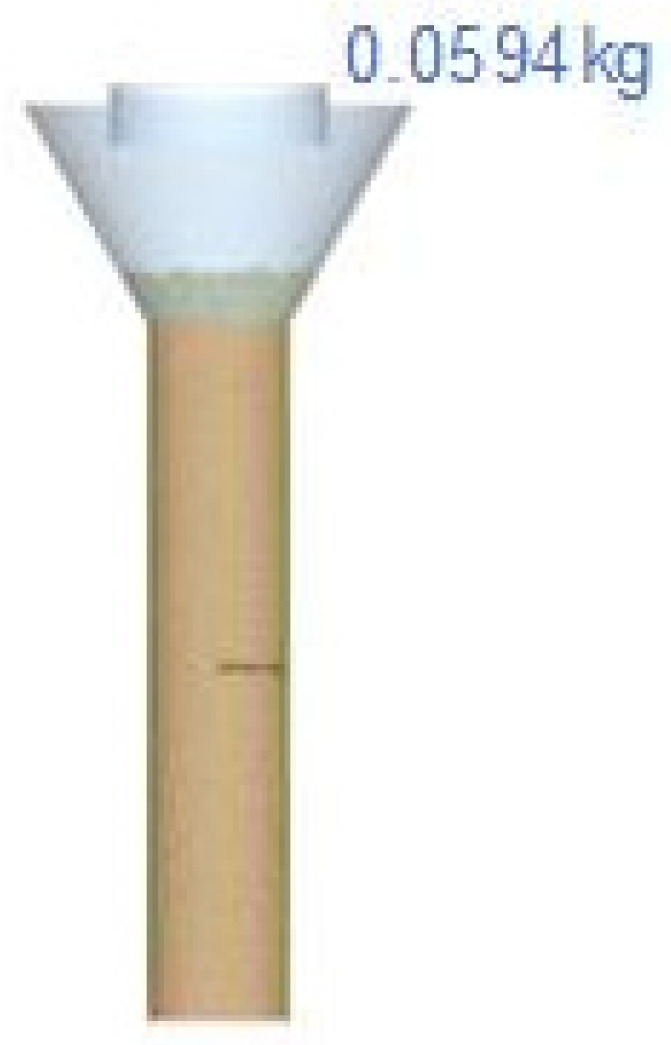
Direct bulk density measurement in test simulation.

Bulk density is directly related to the contact parameters. In the simulation of the bulk density measuring device, the process starts with the empty cylinder (inner diameter of 38 mm and 158.65 mm high). This cylinder is filled with an amount of powder. Subsequently, we wait the necessary time to stabilize the gravity compaction. The following step is to measure the weight of powder that fits in the bottom cylinder (Figure 12). The weight of powder inside the cylinder (m_VB_) is measured directly by the EDEM software.

##### Hollow Cylinder Simulation

**Figure 13:**
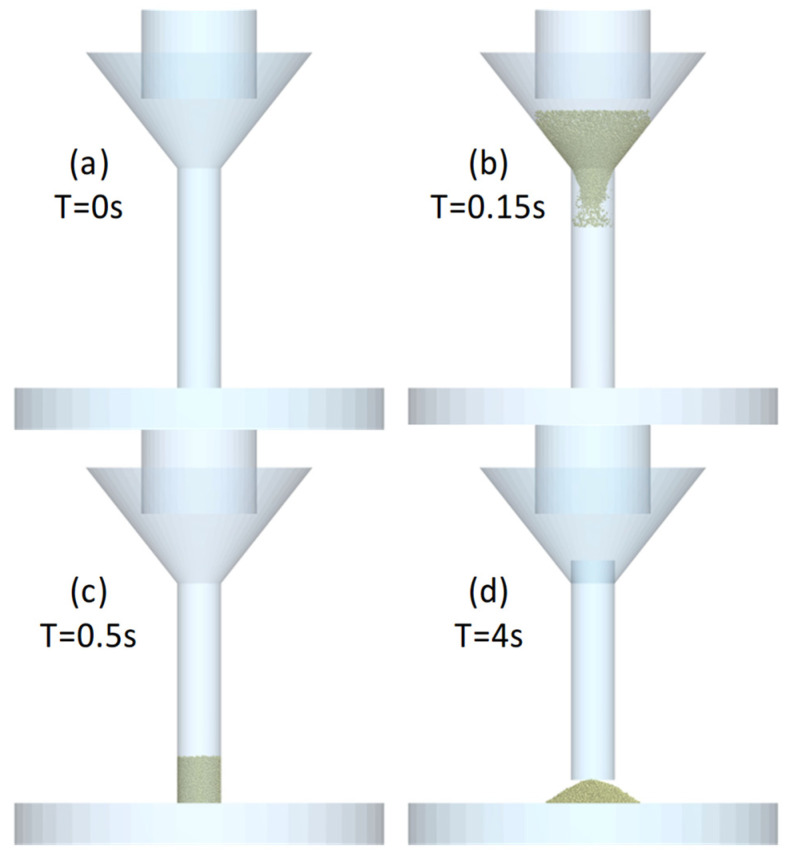
Hollow cylinder process in software for four different time steps.

In the simulation of the hollow cylinder test, we have three parts: a funnel, a hollow cylinder (38 mm in diameter and 200 mm high), and a flat surface. The simulation process (Figure 13) starts with the empty cylinder on the flat surface and the funnel on the cylinder. An amount of powder is generated, filling the cylinder. Subsequently, we wait for 3 s to give enough time so that the powder can stabilize. After that, the cylinder goes up 100 mm with a constant speed of 0.01 m/s, and we wait again the time necessary to stabilize the powder dropping. Now we can measure the angle with the horizontal (α_SHC_) using an image processing method (explained in Section 2.4. angle measurement process).

##### Ledge Box Simulation

**Figure 14:**
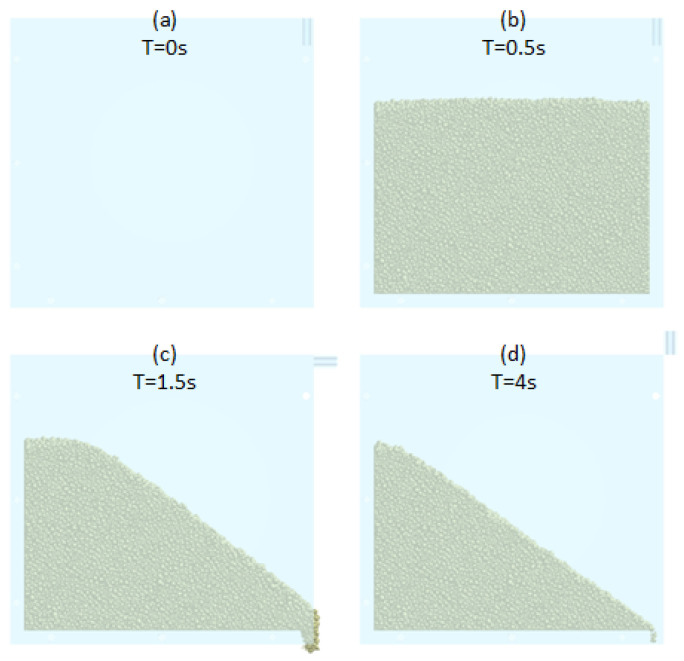
Ledge box process in software for four different time steps.

For the Ledge box simulation, the box (75 × 100 × 16 mm inner dimensions) is filled with the necessary powder mass. After that, we wait 1 s for the powder stabilization. Then, the right wall is opened with a velocity of π rad/s (Figure 14). Now, after powder stabilization is possible to measure the static friction angle (ϕ_VLB_) using an image processing method (explained in Section 2.4. angle measurement process).

##### Draw Down Simulation

**Figure 15:**
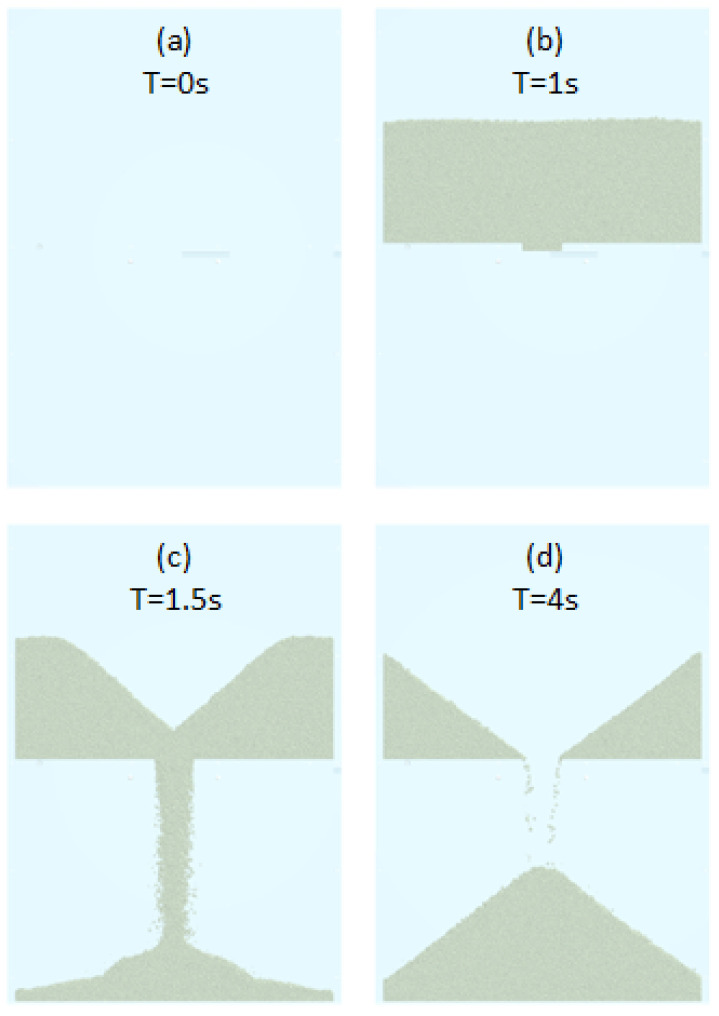
Drawdown process in software for four different time steps.

In the case of the drawdown test (the inner dimension box was 125 × 60 × 12 mm, and the gat width was 6.5 mm), we start in the same way as in the other tests, filling and waiting for the powder stabilization. After waiting 1 s, the gate is opened with a velocity of 1 m/s (Figure 15). This test takes a long time to simulate the powder drop until the stabilization. Therefore, we adjusted the total test time to each case study. When the test is finished, we can measure directly in the software the weight of powder in the bottom part (m_VDD_), but we need an image processing procedure to measure the angle of repose (α_VDD_) in the bottom part and the static friction angle (ϕ_VDD_) in the upper section (method explained in Section 2.4. angle measurement process).

### 2.4. Angle Measurement Process

**Figure 16:**
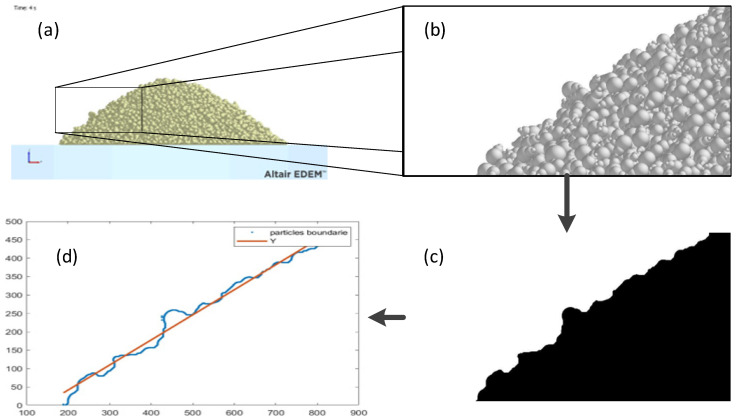
Image processing method: initial picture (**a**), sectioned part (**b**), binarized section (**c**), and boundary with fitted equation (**d**).

For the angle measurement, an image processing method was developed for the Matlab environment. This method starts with a picture of the finished test that we are analyzing. This picture is sectioned and focused on the corresponding pile of powder. This section is binarized (transformed to black and white), and the boundary contours of the particle pile are obtained. By the least squares fitting method, we obtained the equation that fits with the boundary (Figure 16).

With this last equation we are able to calculate the angle using the slope (m) and Equation (10).

(10) $$\alpha =\frac{\tan^{-1}\left|m\right|\cdot 180}{\pi }$$

### 2.5. Optimization with Genetic Algorithms

Genetic algorithms mimic the law of natural selection in nature [50,51,52], but in a virtual environment, allowing for a robust search, in this case to find the optimal combination of design variables so that the simulation results match well with the experimental results. This algorithm starts with a 100 individual generation, which means 100 combinations of variables created randomly. Afterward, the fitness functions are evaluated by the algorithm for each design. In this case, this evaluation is carried out by the use of an interpolation method (Kriging metamodel) [53,54] for the different response variables. These metamodels are created using the obtained data in a group of previous experiments. An exponential correlation model and a polynomial regression model are used in the Kriging metamodel. However, the polynomial regression is able to change the order from 2 to 0 depending on the available data. The chosen order depends on the number of samples and the distribution of them, so a 0-order polynomial is easier to calculate and needs fewer samples, but it is also less accurate.

This is the reason why the algorithm includes a loop that starts using a 2nd-order regression model for the prediction of each response variable; in case the meta-model fails, the order of the regression is downgraded to 1 automatically; if it fails again, it is downgraded to a zero-order regression model. Therefore, this loop guarantees the use of the best regression model possible with the available data.

For the application of this algorithm, an initial database is necessary. These data are generated using the Latin Hypercube design of experiments [51] but with some modifications. In a first step, a combination with all the variables with the minimum values, a second combination with all the variables in the middle of its range, and a third combination with all the variables at the maximum of its range are generated. This implies three different designs. Subsequently, the Latin Hypercube algorithm is applied, adding n points, where n is the number of variables (10 in this work). Therefore, the algorithm divides the range of each variable into equal parts, chooses a random value of each variable, and deletes the corresponding range for each variable in the list of possible values to use in the next variable combination. This process is repeated until adding n points. This means the optimization process begins with a total of 3 + n sample points (13 initial combinations in our case).

As the Kriging metamodel achieves better results when interpolating rather than extrapolating, the Latin Hypercube algorithm was modified to choose the minimum or maximum value of each range when sampling points are on extreme ranges. This way, the data inside the extreme ranges were displaced to the border of the search space, thus promoting interpolation over extrapolation.

The genetic algorithm, implemented in MATLAB (R2022a), allows the search of the combination of coefficients that results in the minimum error between the simulated test and the real results.

### 2.6. Calibration Process

The calibration process started with the limit selection for each variable and establishing the fixed data. Those limits were defined according to the limits found in the bibliography and the information provided by Altair EDEM (2023) software (Table 4). Those parameters depend on the particle material, the boundary material, and the kind of experiment chosen as reference.

**Figure 17:**
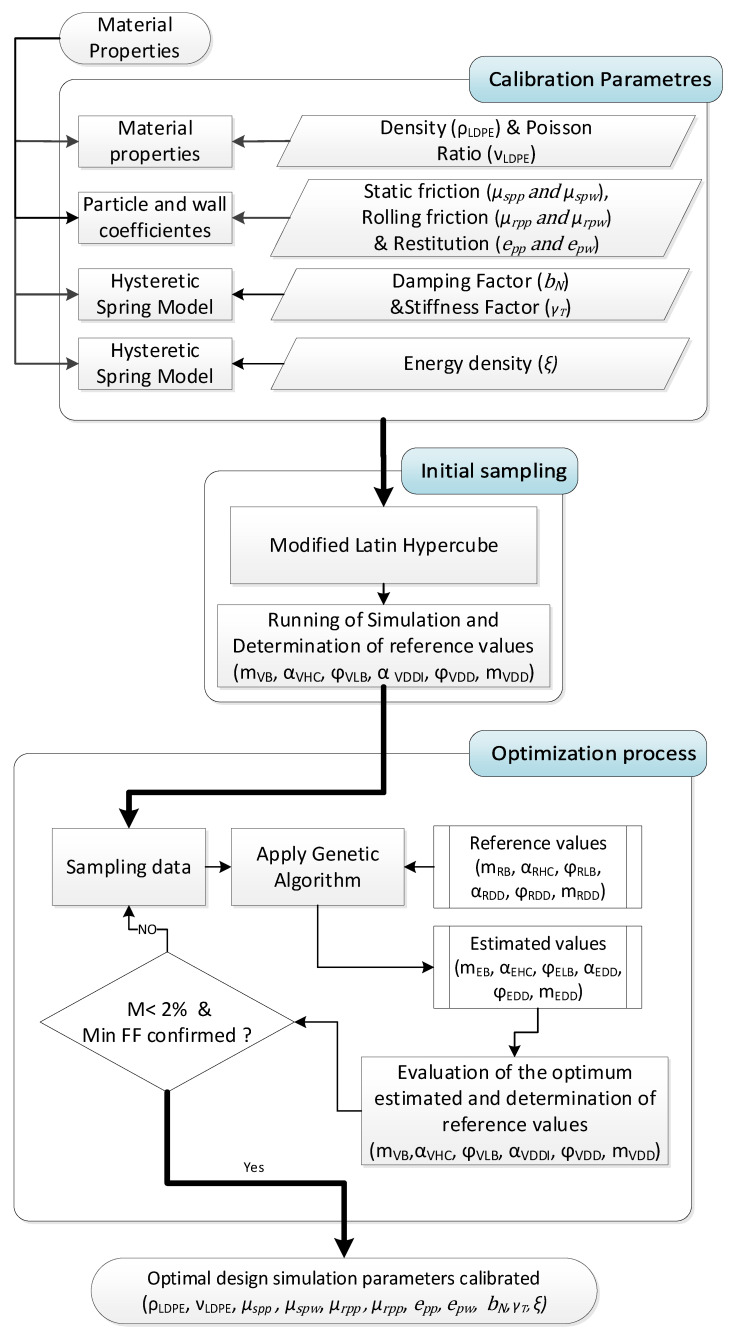
Flow chart calibration process.

During the calibration process, the flow chart shown in Figure 17 was used.

As mentioned before, the variables to optimize or calibrate in this study were the powder density (ρ_LDPE_), the Poisson’s Ratio (ν_LDPE_), coefficients off restitution ($e_{pp}$ and $e_{pw}$), coefficients off static friction (${µ}_{spp}\;and\;{µ}_{spw}$), the coefficients off rolling friction (${µ}_{rpp}$ and ${µ}_{rpw}$), the damping factor ($b_{N}$), the stiffness factor ($\gamma_{T}$) and the energy density (ξ). The restitution, static friction, and rolling friction factors are calibrated both for the particle-particle interaction and the particle-wall interaction.

On the other hand, as previously mentioned, the response variables (result values to compare between estimated results by the genetic algorithm and simulations) were the estimated bulk mass (m_EB_), the estimated static angle of repose (α_EHC_), the estimated shear angle (ϕ_ELB_), the DD estimated static angle of repose (α_EDD_), the DD estimated shear angle (ϕ_EDD_), and the estimated dropped mass (m_EDD_).

**Figure 18:**
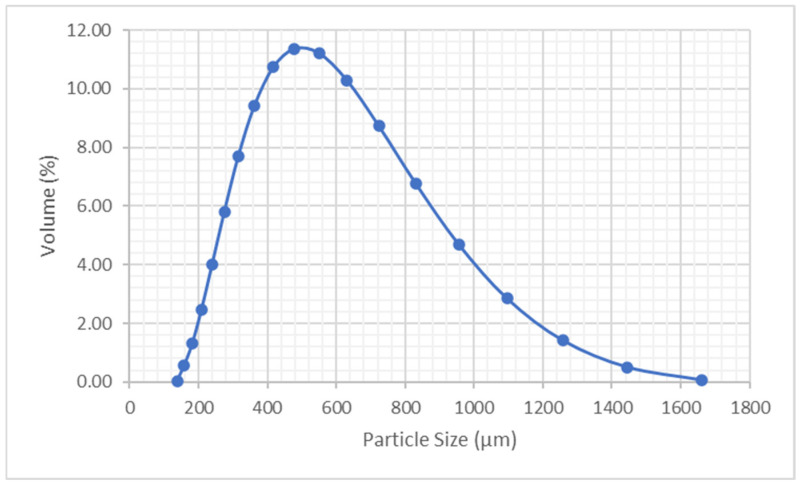
Particle size distribution of LDPE powder.

The Mean Absolute Percentage Error (MAPE, or M in Figure 18) between the estimated values during the optimization (or the simulated responses) and the reference results from the experimental tests was used as a fitness function (FF) for the optimization (and also as adjusting stop criterion), since it measures the accuracy of the predicted data, in percentage, attending to Equation (11).

(11) $$MAPE=100/n_{r}\sum_{t=1}^{n_{r}}\left|\left(A_{t}/F_{t}\right)/A_{t}\right|$$

where *n_r_* is the number of response variables (6 in our case), *A_t_* is the reference value (the real result from the experimental test), and *F_t_* is the predicted response by the genetic algorithm during optimization (or the simulation data for the stop criterion), all of this for each response variable (*t*).

Once the genetic algorithm obtains the optimal design (design with the minimum estimated MAPE), the selected combination of design variables is introduced into the four reference virtual test simulations. The results of these simulations are used to evaluate the MAPE between the simulated results and the experimental reference values. The calibration process finishes in the moment that the minimum estimated MAPE is obtained (using the estimated values of the genetic algorithm) and the MAPE between the simulated results of the selected optimum and the corresponding experimental values is lower than 2%. If these criteria are not met, the genetic algorithm is run again, but the results of the simulations of the estimated optimal design in the previous iteration are added as an additional sampling point.

Since sometimes the stopping criteria are not reached after a great number of iterations, every 10 iterations without reaching the stopping criterion, the results of those last 10 iterations are analyzed. If the trend of these results is towards the objective (2% MAPE), 10 more iterations are performed. In case the results start to fluctuate without a tendency to improve, the best result in the last 10 iterations will be assumed to be the optimum.

To ensure that this procedure remains universal and applicable across any Discrete Element Method (DEM) software, the process of reading and inputting data into the simulation software is performed manually, following these sequential steps.

We manually input the desired set of parameters into the EDEM configuration and launch the simulation.

Once the simulation concludes, we read the resulting mass outputs and capture an image of the results to measure the angles, as described in Section 2.4.

These resulting data are then manually entered into a spreadsheet (e.g., Excel), where the optimization criteria (Mean Absolute Percentage Error (MAPE) <2% and Fill Factor (FF) confirmation) are calculated.

Should the stopping criteria not be met, we copy the table containing all previously simulated parameter combinations and their objective results into our genetic algorithm (MATLAB).

With this new information, we execute the genetic algorithm once more.

The new parameter combination suggested by the algorithm is then used to manually configure a new batch of simulations, and the cycle recommences.

## 3. Results and Discussion

### 3.1. Real Environment

#### 3.1.1. Powder Preparation and Shape Characterization

After the micronization, the diameter distributions obtained in the granulometries were the ones shown in Figure 18, with a surface-weighted mean D (3,2) of 472.997 μm and a volume-weighted mean D (4,3) of 579.025 μm.

The particles were divided into 19 groups (according to the particle size distribution) to measure the average aspect ratio for each group. The results are shown in Table 5. The third column shows the truncated groups (from group 1 to group 6) and the equivalent aspect ratio. These groups were chosen because they represent less than 20%, of the total volume. If the group number 7 were added, the truncated volume would exceed that limit of 20% affecting the performance of the powder in the simulations. Finally, 14 groups of particles were represented in the simulations.

#### 3.1.2. Determination of the Bulk Density, Tap Density, and Flowability

Eight replicates of the flowability test were carried out. The results (including the Haussner and Carr Indexes) are shown in Table 6.

Based on these results, the powder is determined as to be “Good/free flow” and compatible with the use of the hysteretic spring contact model.

#### 3.1.3. Experimental Reference Tests

Three iterations of each experimental reference test were performed, and the benchmark data were recollected. Table 7 shows a summary of the results.

It is shown some differences in the angle measures in each iteration, something that affects all tests. This could be an effect of different variables, such as the humidity absorbed by the powder, the static electricity of the acrylic, or the random effect of the natural compaction during the refill of the machine or the use process itself. For that reason, three iterations are made, and the data used as reference is the average of those iterations, which is given by the average of the two sides measured.

### 3.2. Virtual Environment

#### 3.2.1. Particle Shape Design

**Figure 19:**
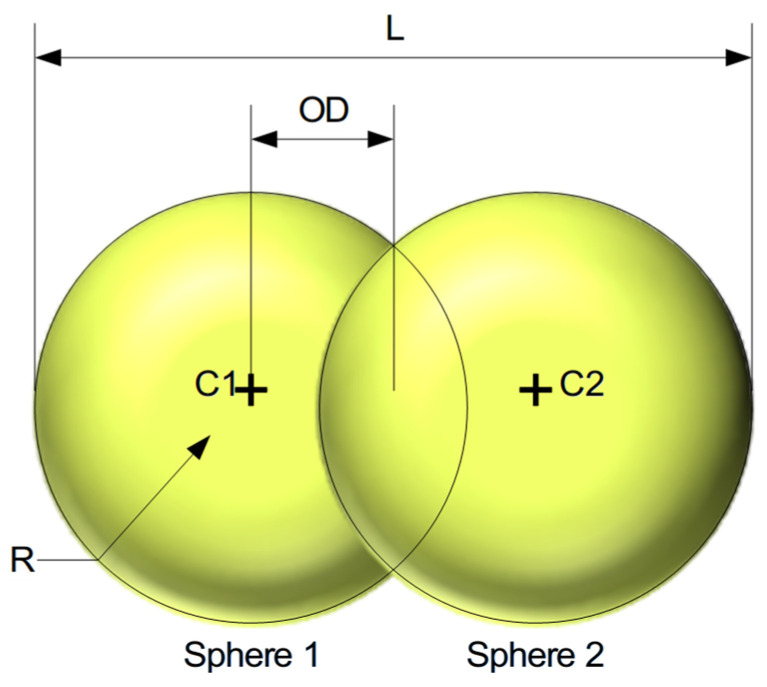
Representation of the particle shape used for the study.

The particles were represented with two overlapped spheres (Figure 19). According to Section 3.1.1, the particles were grouped by diameter into 14 different groups (the first group being the set of the six initial smallest groups), each of them with the corresponding aspect ratio. For representing this in the virtual environment, two spheres were used, defining their diameter as the average width of the measured particles and adjusting the overlapped distance (OD) to match the measured length (and consequently) the aspect ratio. Table 8 shows the final dimensions and OD between spheres.

#### 3.2.2. Calibration Process

The fitness function for the optimization process is defined with the information from, which is represented in Equation (12).

(12) $$FF=M=\frac{100}{6}(\left(\left|\frac{35.6-obj(1)}{35.6}\right|\right)+\left(\left|\frac{60.7-obj(2)}{60.7}\right|\right)+\left(\left|\frac{55.4-obj(3)}{55.4}\right|\right)\;\;\;\;\;\;\;+\left(\left|\frac{38.9-obj(4)}{38.9}\right|\right)+\left(\left|\frac{3.3-obj(5)}{3.3}\right|\right)+\left(\left|\frac{59.4-obj(6)}{59.4}\right|\right))$$

During the optimization, some combinations resulted in a powder with a flowability so poor or a resiliency so high that any measurement in the virtual environment was impossible. For this reason, objectives 7 (for the Ledge box test) and 8 (for the drawdown test) were also introduced as restrictions. When it was impossible to measure the result of the simulation test, a 0 was assigned; when the possibility of measuring was perfect, a 1 was assigned; and when it was possible to measure, but the powder was not stable during the time proposed, a 0.5 was assigned. The limits for these restrictions were established at 0.5, so values of 0.5 or higher were considered feasible.

With this, the modified Latin Hypercube algorithm was applied for a total of 14 different points. In this case, the variables corresponding to the initial sampling points are shown in Table 9, and the results in Table 10.

With these sampling data, the optimization algorithm was run. The estimated optimum was simulated in the reference tests to obtain the simulation outputs and use these results to assess the stop criteria. If the stop criteria were not achieved, the data were added to the sampling data and the optimization applied again. Finally, after 85 iterations, the genetic algorithm reached the stop criteria.

The best combination of variables is shown in Table 11, and the simulation results for that combination in Table 12.

To achieve this objective, almost twice as many iterations were performed as in the previous literature. However, given the low minimum error percentage achieved in the FF, we can see that the scaling of the powder and the manipulation of some particle diameters did not affect its performance in the different tests.

**Figure 20:**
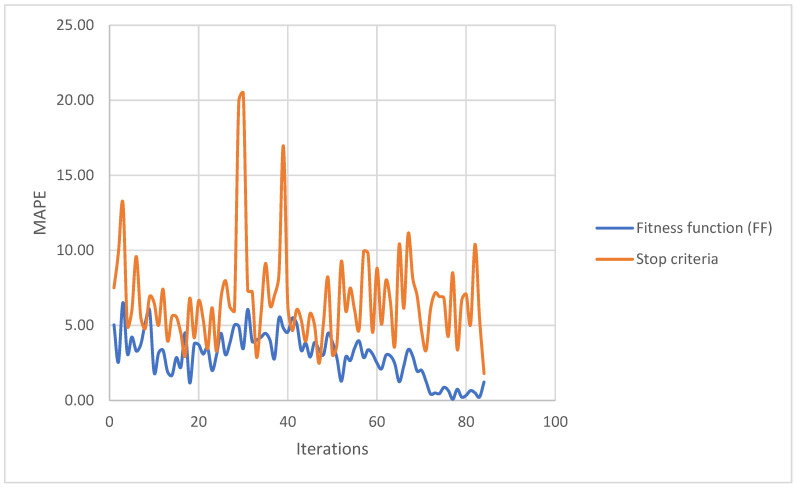
Trend of the results of the calculated equations (MAPE) for the different iterations.

Figure 20 shows how the objective function is progressively achieved until it reaches a point where it begins to fluctuate, and it is not until the stopping criterion is met that we end the iterations. All data recollected during the iterations could be shown on the Supplement Material (Table S1).

## 4. Conclusions

Using a powder calibration procedure for DEM (Discrete Element Method) based on the application of genetic optimization algorithms, which have undergone simulation acceleration procedures known as scaling and scalping, a combination of material physical variables has been achieved. This combination enables bringing the simulation results closer to those obtained in real experiments.

After a total of 85 iterations, the results of the genetic algorithm reached the stop criteria. This means that the error of the prediction by the genetic algorithm was less than 2% with respect to the simulation results and the error of the fitted function—comparing the simulation results, with the real experiments—is the minimum obtained (2.98%). The best result, as well as the rest of the iterations, could be found in the Supplementary Material (Table S1).

As suggested by reference [59], calibration results may exhibit ambiguity, especially when calibrating more parameters than available reference values. This issue may be solved by using surrogate models and population-based optimization algorithms, as proposed in [60]. However, in this reference [60], the authors used fewer calibration parameters than objective references, leaving for future work the analysis of the suitability of the developed algorithm for the calibration of more parameters. In this regard, we must highlight the following:

The application of genetic algorithms combined with the Kriging metamodel and design of experiments has successfully demonstrated the capability to converge on a result with a very small mean absolute percentage error (MAPE), even when using more calibration parameters than objective references. Furthermore, upon examining the combinations of parameters that yield the best MAPE values in our study, we observe that several of them are coincident. We can therefore conclude that the resulting optimal combination is not ambiguous and appears to be unique or near-unique.

On the other hand, it was observed a considerable increase in the iteration number compared to previous works [20], but this may be due to high number of variables used. In previous studies, six variables were used, which after 51 iterations resulted in a ratio of 8.5 iterations per variable, whereas in this study, a total of 11 variables were used, calibrated in 85 iterations, resulting in 7.7 iterations per variable. This leads us to conclude that the method works just as fast as previous studies and approximately proportional to the number of variables. It also demonstrates that the genetic algorithm optimization is not affected by the other speed-up methods.

This opens the possibility of calibrating more complex contact models with more variables and a better approximation to reality.

The scaling-up method sometimes is limited by the experiment’s dimensions, so it is something to take into account when the experiment is designed. For example, the gate width on the drawdown test could produce stuck powder if we scale up too much. To avoid this, we can consider how much the particle will be scaled and design the experiment dimensions accordingly.

Regarding the scalping method, no contraindications to its use have been found.

In light of the preceding analysis, it has been successfully demonstrated that the combined use of both methods is indeed feasible. However, to achieve optimal efficiency, it has been observed that the best practice is to first employ the scalping method up to the limiting size established in the literature. Once this initial simplification is performed, we then proceed to scale all the resulting particle groups.

Should the particles be overscaled, we might consequently be compelled to also scale the boundaries of the computational domain. Conversely, insufficient scaling will result in an unacceptably excessive number of particles requiring simulation. It is therefore imperative that future investigations meticulously analyze the optimal balance between the degree of particle scaling and the necessity of maintaining a computationally reasonable particle count.

Another matter we intend to study in future work is the addition of dynamic flow tests. These tests could significantly improve the fidelity of the simulation’s approximation to the material’s real-world behavior [61,62]. Furthermore, they would help to accelerate the calibration process by incorporating additional objective functions into the fitness function assessment without increasing the number of calibration variables.

In addition, two overlapping spheres were used to represent the particles in this study. However, it has been shown that the shape of the particle can affect the macroscopic results of the powder [46,63,64]. Future research will analyze how this improved representation affects the calibration speed of the procedure.

## Figures and Tables

**Table 1:** Haussner ratio and Carr’s index coefficients [23].

Flow Character	Hausner Ratio	CI%
Excellent/very free flow	1.00–1.11	≤10
Good/free flow	1.12–1.18	11-15
Fair	1.19–1.25	16–20
Passable	1.26–1.34	21–25
Poor/cohesive	1.35–1.45	26–31
Very Poor/very cohesive	1.46–1.59	32–37
Very, very poor/Approx. non-flow	>1.6	>38

**Table 2:** Hert–Mindlin (no slip) contact model equations.

**Normal Force**	$F_{N}=\frac{4}{3}E_{eq}\sqrt{R_{eq}}\delta_{n}^{3/2}$
**Tangential Force**	$F_{T}=-min\left\{K_{T},\delta_{T},{µ}_{s}\left|F_{N}\right|\right\}\cdot t$
**Normal and tangential damping force**	$F_{N,T}^{d}=-2\sqrt{\frac{5}{6}}\frac{\ln e}{\sqrt{\ln^{2}e+\pi^{2}}}\sqrt{K_{N,T}m_{eq}}V_{N,T}^{rel}$
**Normal and Tangential Stiffness**	$K_{N}=2E_{eq}\sqrt{R_{eq}\delta_{N}}\;;\;K_{T}=8G_{eq}\sqrt{R_{eq}\delta_{N}}$
**Equivalent Young modulus**	$\frac{1}{E_{eq}}=\frac{\left(1-V_{i}^{2}\right)}{E_{i}}+\frac{\left(1-V_{j}^{2}\right)}{E_{j}}$
**Shear modulus**	$G_{eq}={\left[\frac{2\left(2-V_{i}\right)\left(1+V_{i}\right)}{E_{i}}+\frac{2\left(2-V_{j}\right)\left(1+V_{j}\right)}{E_{j}}\right]}^{-1}$
**Equivalent Radio**	$\frac{1}{R_{eq}}=\frac{1}{R_{i}}+\frac{1}{R_{j}}$
**Equivalent Mass**	$m_{eq}={\left(\frac{1}{m_{i}}+\frac{1}{m_{j}}\right)}^{-1}$
**Rolling friction**	$\tau =-{µ}_{r}F_{N}R$

where N y T mean Normal and Tangential, respectively, i and j correspond to each particle interacting, *e* is the restitution coefficient, ${µ}_{s}$ represents the static friction coefficient, ${µ}_{r}$ is the rolling friction coefficient, and V the relative speed of the particle.

**Table 3:** Hysteretic spring contact model equations.

**Normal Force**	$F_{N}=\left\{\begin{array}{ccc} K_{1}\delta_{N} & for\;loading & \left(K_{1}\delta_{N}<K_{2}(\delta_{N}-\delta_{0})\right)\\ K_{2}(\delta_{N}-\delta_{0}) & for\;unloading/reloading & \delta_{N}>\delta_{0}\\ 0 & for\;unloading & \delta_{N}\leq \delta_{0} \end{array}\right.$
**Tangential Force**	$F_{T}=-min\left(\gamma_{T}K_{1}\delta_{T}+F_{T}^{d},{µ}_{s}F_{N}\right)$
**Loading Stiffness**	$K_{1}=5R_{eq}min\left(Y_{1},Y_{2}\right)$
**Unload Stiffness**	$K_{2}=\frac{K_{1}}{e^{2}}$
**Material Yield Strength**	$Y=\frac{4}{15}\frac{E}{\sqrt{R}}\frac{1}{70}$
**Overlap**	$\delta_{0}=\left\{\begin{array}{ccc} \delta_{N}\left(1-\frac{K_{1}}{K_{2}}\right) & for\;loading & \left(K_{1}\delta_{N}<K_{2}(\delta_{N}-\delta_{0})\right)\\ \delta_{0} & for\;unloading/reloading & \delta_{N}>\delta_{0}\\ \delta_{N} & for\;unloading & \delta_{N}\leq \delta_{0} \end{array}\right.$
**Normal damping force**	$F_{N}^{d}=-b_{N}\sqrt{\frac{4mK}{1+{\left(\frac{\pi }{\ln e}\right)}^{2}}}v_{N}^{rel}$
**Tangential damping force**	$F_{T}^{d}=-\sqrt{\frac{4m\gamma_{T}K}{1+{\left(\frac{\pi }{\ln e}\right)}^{2}}}v_{T}^{rel}$
**Moment effect**	$M=r_{con}\cdot F_{T}$
**Rotation moment**	$M_{r}=-{µ}_{r}{\cdot F}_{N}\cdot r_{con}\cdot \lambda_{\theta }$
**Resultant moment**	$M_{res}=M+M_{r}$

where δ is the overlap, $\delta_{0}$ is the residual overlap, e is the restitution coefficient, $b_{N}$ is the damping factor, $\gamma_{T}$ is the stiffness factor, ${µ}_{s}$ the static friction coefficient, $r_{con}$ is the distance between the contact point and the center of mass, ${µ}_{r}$ is the rolling friction coefficient, and $\lambda_{\theta }$ is the unit vector of angular velocity (θ).

**Table 4:** Variable limits.

		Units	Min	Max	Reference
LDPE	ν_LDPE_	Dimensionless	0.2	0.5	Altair Edem software
	ρ_LDPE_	kg/m^3^	450	1400	Altair Edem software
	G	Pa	1.00 × 10^7^		Altair Edem software
Acrilic	ν*_Acrilic_*	Dimensionless	0.4		[49]
	*ρ_Acrilic_*	kg/m^3^	1385		[49]
	*G*	Pa	1.60 × 10^10^		[49]
Particle-Particle	*e_pp_*	Dimensionless	0.1	0.78	[55,56]
	*μ_spp_*	Dimensionless	0.1	0.95	[57,58]
	*μ_rpp_*	Dimensionless	0	0.25	[20,34]
Particle-Wall	$e_{pw}$	Dimensionless	0.1	0.78	[55,56]
	*μ_spw_*	Dimensionless	0.1	0.95	[57,58]
	*μ_rpw_*	Dimensionless	0	0.2	[13]
Particle-Particle HSCM	*b_N_*	Dimensionless	0	0.1	Altair Edem software
	*γ_T_*	Dimensionless	0.67	1	[40]
Particle-Particle LCCM	*ξ*	J/m^3^	1	8000	[36]

**Table 5:** Particles aspect ratio.

Group of Particles	Average Aspect Ratio	Truncated Group Aspect Ratio
1	1.75	1.64
2	1.70	
3	1.42	
4	1.59	
5	1.54	
6	1.82	
7	1.25	1.25
8	1.74	1.74
9	1.46	1.46
10	1.32	1.32
11	1.20	1.20
12	1.30	1.30
13	1.43	1.43
14	1.63	1.63
15	1.08	1.08
16	1.48	1.48
17	1.48	1.48
18	1.48	1.48
19	1.48	1.48

**Table 6:** Flowability results.

	Vb (cm^3^)	Vt (cm^3^)	ρb (g/cm^3^)	ρt (g/cm^3^)	Ρb (kg/cm^3^)	ρt (kg/cm^3^)	H	Ci%
1	54.5	44.5	0.367	0.449	367	449	1.22	18
2	52.5	47	0.381	0.426	381	426	1.12	10
3	56	46.75	0.357	0.428	357	428	1.20	17
4	55.5	47	0.360	0.426	360	426	1.18	15
5	55	47	0.364	0.426	364	426	1.17	15
6	57	47.5	0.351	0.421	351	421	1.20	17
7	54	47	0.370	0.426	370	426	1.15	13
8	52.5	47	0.381	0.426	381	426	1.12	10
Average (standard deviation)	54.6 (1.60)	46.71 (0.92)	0.366 (0.01)	0.428 (0.01)	366 (10.76)	428 (8.77)	1.17 (0.04)	14 (2.90)

**Table 7:** Real benchmark results.

			Real Measures			
Test	Variable	Measure	It1	It2	It3	Total Average
HC	α_RHC_ (^o^)	Left	30.8	38.6	32.3	35.6
		Right	35.8	31.8	44	
		Average iteration	33.3	35.2	38.15	
LB	ϕ_RLB_ (^o^)	Unique	50	66.3	65.7	60.7
DD	ϕ_RDD_ (^o^)	Left	45.2	56.1	55.3	55.4
		Right	52	53.3	70.3	
		Average iteration	48.6	54.7	62.8	
	α_RDD_ (^o^)	Left	43.4	34.9	40.7	38.9
		Right	39.3	38.8	36.5	
		Average iteration	41.35	36.85	38.6	
	m_RDD_ (gr)	Direct	3.3	3	3.7	3.3
Bulk density	m_RB_ (gr)	Direct	60.14	59.04	58.98	59.4

**Table 8:** Particle dimensions.

	(μm)		
Particle Group	Radio (R)	Lenth (L)	OD
1–6	138	501	113
7	158	394	39
8	182	632	134
9	208	610	96
10	239	630	76
11	275	660	55
12	315	822	95
13	362	1034	155
14	416	1360	264
15	477	1033	39
16	548	1625	264
17	629	1866	304
18	723	2143	349
19	830	2460	400

**Table 9:** Initial sampling.

	Particle		Particle-Particle			Particle-Walls			P-P HSMC		P-P LCMC
	Ρ_LDPE_	Ν_LDPE_	*e_pp_*	*μ* *_spp_*	*μ* *_rpp_*	*e_pw_*	*μ* *_spw_*	*μ* *_rpw_*	*b_N_*	*γ_T_*	*ξ*
EP1	450	0.2	0.1	0.1	0	0.1	0.1	0	0	0.67	1
EP2	925	0.35	0.44	0.53	0.13	0.44	0.525	0.1	0.05	0.835	4000
EP3	1400	0.5	0.78	0.95	0.25	0.78	0.95	0.2	0.1	1	8000
EP4	623	0.26	0.207	0.479	0.044	0.503	0.739	0.085	0.054	0.776	5959
EP5	765	0.43	0.553	0.950	0.077	0.230	0.950	0.015	0.079	0.813	2543
EP6	450	0.25	0.503	0.394	0.064	0.215	0.345	0.039	0.085	0.949	1099
EP7	1284	0.20	0.618	0.726	0.058	0.632	0.687	0.033	0.070	0.914	5181
EP8	978	0.45	0.333	0.711	0.017	0.290	0.583	0.068	0.025	0.872	8000
EP9	1400	0.37	0.460	0.560	0.100	0.538	0.499	0.100	0.039	1.000	4037
EP10	587	0.42	0.780	0.211	0.036	0.365	0.307	0.018	0.031	0.906	2936
EP11	1208	0.35	0.238	0.100	0.086	0.433	0.212	0.079	0.000	0.735	5061
EP12	1130	0.32	0.100	0.617	0.023	0.100	0.411	0.000	0.012	0.846	1475
EP13	942	0.29	0.712	0.318	0.045	0.780	0.100	0.048	0.063	0.670	1
EP14	819	0.5	0.398	0.847	0.000	0.710	0.847	0.055	0.100	0.719	6788

**Table 10:** Initial sampling results.

	Simulation Results								Objective (MAPE)
	obj(1)	obj(2)	obj(3)	obj(4)	obj(5)	obj(6)	obj(7)	obj(8)	
	α_HC_	ϕ_LB_	ϕ_DD_	α_DD_	m_DD_	m*_VB_*	V_DD_	V_LB_	
EP1	3.3	14.6	13.3	21	7	50.8	1	0.5	68.86
EP2	34.4	47.6	51.2	24	2.9	92.3	0.5	0.5	23.18
EP3	40.6	60.2	52.2	21.6	1.8	124.5	0.5	0.5	36.81
EP4	29.2	42.8	40.8	28.5	4.7	65.2	0.5	0.5	25.2
EP5	33.2	43.4	50.2	24.9	3.7	70	0.5	1	18.22
EP6	35.4	43	44.3	36.1	5.4	45.4	1	0.5	23.73
EP7	28.8	46.3	48.4	38.2	2.6	121.3	1	1	30.56
EP8	25	40.8	42.6	25	3.7	102.8	1	0.5	34.23
EP9	28.5	51.2	45	18.1	2.3	140.1	0.5	1	45.76
EP10	22.2	26.5	31.6	24.1	5.7	60.8	1	0.5	41.38
EP11	17.4	22.6	23.3	28.2	4.5	134.3	1	0.5	60.07
EP12	16.4	44.1	34.9	12.6	4	123.4	0.5	0.5	52.26
EP13	11.1	24.3	22.1	28.5	5.6	94.9	1	0.5	57.23
EP14	24.9	31	37.7	23.9	4.5	83.6	0.5	0.5	37.53

**Table 11:** Final combination.

	Particle		Particle-Particle			Particle-Walls			P-P HSMC		P-P LCMC
	ρ	ν	*e*	*μ* *_s_*	*μ* *_r_*	*e*	*μ* *_s_*	*μ* *_r_*	*b_N_*	*γ_T_*	*ξ*
Op85	664	0.20	0.662	0.526	0.149	0.463	0.950	0.107	0.081	1.000	5750

**Table 12:** Final simulation results and comparison with the experimental results.

	obj(1)	obj(2)	obj(3)	obj(4)	obj(5)	obj(6)	FF	Objetive (MAPE)
	α_HC_	ϕ_LB_	ϕ_DD_	α_DD_	m_DD_	m*_VB_*		
Op85	35.0	51.0	56.9	37.0	3.3	62.7	2.98	1.8
Experiments	35.6	60.7	55.4	38.9	3.3	59.4		

## Data Availability

The original contributions presented in this study are included in the article. Further inquiries can be directed to the corresponding authors.

## Supplementary Materials

The following supporting information can be downloaded at: https://www.mdpi.com/article/10.3390/polym17202748/s1, Table S1: Iterations results table.
