# Supplementary material for: Development of a Non-Spherical Polymeric Particles Calibration Procedure for Numerical Simulations Based on the Discrete Element Method
Source: Polymers (Basel). 2025 Oct 14;17(20):2748. doi: 10.3390/polym17202748 (PMC12566918; doi:10.3390/polym17202748)
Supplement: Supplementary file 1 [file polymers-17-02748-s001.zip › polymers-3869613-supplementary.pdf]

	Variables											Simulation results						Objetive -FF (MAPE)								
	Particle		Particle-Particle			Particle-Walls			PP HSMC		PP LCMC	obj(1)	obj(2)	obj(3)	obj(4)	obj(5)	obj(6)									
	ρ	ν	e	μs	μr	e	μs	μr	bN	γT	ξ	αHC	ϕLB	ϕDD	αDD	mDD	Bm									
EP1	450	0.20	0.100	0.100	0.000	0.100	0.100	0.000	0.000	0.670	1	3.3	14.6	13.3	21	7.0	50.8	68.34								
EP2	925	0.35	0.440	0.525	0.125	0.440	0.525	0.100	0.050	0.835	4000	34.4	47.6	51.2	24	2.9	92.3	20.20								
EP3	1400	0.50	0.780	0.950	0.250	0.780	0.950	0.200	0.100	1.000	8000	40.6	60.2	52.2	21.6	1.8	124.5	40.04								
EP4	623	0.26	0.207	0.479	0.044	0.503	0.739	0.085	0.054	0.776	5959	29.2	42.8	40.8	28.5	4.7	65.2	22.89								
EP5	765	0.43	0.553	0.950	0.077	0.230	0.950	0.015	0.079	0.813	2543	33.2	43.4	50.2	24.9	3.7	70.0	15.82								
EP6	450	0.25	0.503	0.394	0.064	0.215	0.345	0.039	0.085	0.949	1099	35.4	43	44.3	36.1	5.4	45.4	21.44								
EP7	1284	0.20	0.618	0.726	0.058	0.632	0.687	0.033	0.070	0.914	5181	28.8	46.3	48.4	38.2	2.6	121.3	27.66								
EP8	978	0.45	0.333	0.711	0.017	0.290	0.583	0.068	0.025	0.872	8000	25	40.8	42.6	25	3.7	102.8	31.98								
EP9	1400	0.37	0.460	0.560	0.100	0.538	0.499	0.100	0.039	1.000	4037	28.5	51.2	45	18.1	2.3	140.1	43.53								
EP10	587	0.42	0.780	0.211	0.036	0.365	0.307	0.018	0.031	0.906	2936	22.2	26.5	31.6	24.1	5.7	60.8	40.09								
EP11	1208	0.35	0.238	0.100	0.086	0.433	0.212	0.079	0.000	0.735	5061	17.4	22.6	23.3	28.2	4.5	134.3	58.94	GA estimation							
EP12	1130	0.32	0.100	0.617	0.023	0.100	0.411	0.000	0.012	0.846	1475	16.4	44.1	34.9	12.6	4.0	123.4	49.82	Estimación de resultados						FF estimated (MAPE)	Stop criteria (MAPE)
EP13	942	0.29	0.712	0.318	0.045	0.780	0.100	0.048	0.063	0.670	1	11.1	24.3	22.1	28.5	5.6	94.9	56.05	obj(1)	obj(2)	obj(3)	obj(4)	obj(5)	obj(6)		
EP14	819	0.50	0.398	0.847	0.000	0.710	0.847	0.055	0.100	0.719	6788	24.9	31	37.7	23.9	4.5	83.6	35.90	αHC	ϕLB	ϕDD	αDD	mDD	Bm		
Op1	637	0.21	0.170	0.950	0.003	0.102	0.407	0.150	0.031	0.880	6291	1	1	1	1	0.1	0.1	97.93	32.0	67.0	60.0	38.9	3.3	59.4	8.83	13648.33
Op2	717	0.20	0.531	0.674	0.134	0.353	0.943	0.088	0.037	0.913	8000	35	46.3	57.2	31.2	3.1	67.2	8.49	34.5	54.3	55.0	32.4	3.3	59.0	5.04	7.51
Op3	729	0.20	0.610	0.950	0.138	0.374	0.842	0.080	0.078	0.864	7066	36.9	51.6	57.4	31.2	3.0	65.7	8.39	32.1	50.4	55.8	38.9	3.3	61.5	2.56	9.86
Op4	633	0.20	0.312	0.597	0.114	0.370	0.884	0.115	0.066	0.973	8000	34.9	46.9	50.5	38.1	3.9	64.0	7.44	42.3	55.1	56.7	36.1	3.3	59.3	6.52	13.15
Op5	652	0.20	0.554	0.660	0.140	0.342	0.815	0.106	0.055	0.972	8000	40.5	51.4	54.6	33.6	3.5	61.5	6.94	37.2	53.3	55.3	36.2	3.3	59.4	3.11	5.00
Op6	692	0.20	0.721	0.629	0.189	0.484	0.839	0.128	0.080	0.956	6895	40.3	53.7	57.9	41.6	3.1	64.1	7.74	42.7	51.7	54.5	38.9	3.3	59.4	4.23	5.97
Op7	694	0.20	0.780	0.950	0.104	0.114	0.766	0.000	0.085	0.915	3869	36.3	46.8	56.5	29	3.5	63.4	8.04	40.1	46.8	55.3	38.6	3.3	59.5	3.30	9.59
Op8	661	0.20	0.632	0.725	0.105	0.250	0.913	0.048	0.089	1.000	5144	35.9	47.9	57	35	3.3	61.1	3.41	42.0	48.5	55.4	38.3	3.3	59.3	3.72	5.57
Op9	625	0.20	0.463	0.454	0.171	0.431	0.950	0.145	0.070	1.000	7996	41.3	50.9	54.7	41.9	3.6	61.2	6.55	42.4	54.8	54.7	39.0	3.3	59.3	5.14	4.78
Op10	688	0.20	0.780	0.488	0.197	0.674	0.950	0.148	0.100	0.998	5698	39	53	56.9	35.9	3.2	65.2	6.53	43.8	56.0	55.4	38.9	3.3	59.1	6.00	6.91
Op11	630	0.20	0.504	0.725	0.125	0.101	0.851	0.060	0.084	0.958	6248	35.8	50.1	52.8	31.5	3.3	59.7	4.23	38.9	49.2	55.4	38.9	3.3	59.8	1.86	6.51
Op12	649	0.20	0.739	0.516	0.191	0.475	0.941	0.132	0.074	0.968	8000	39.9	54.1	53.5	33.2	3.2	61.2	7.48	40.4	50.2	55.0	37.7	3.3	59.9	3.16	5.01
Op13	633	0.20	0.591	0.621	0.152	0.340	0.946	0.105	0.085	0.989	7516	38.8	52.2	58.1	28.8	3.2	59.0	8.06	40.3	51.9	55.5	38.0	3.3	59.3	3.35	7.42
Op14	616	0.20	0.503	0.742	0.133	0.100	0.944	0.062	0.073	0.900	8000	37.3	51.2	54.6	37.9	3.6	57.6	3.95	38.3	48.1	55.4	38.7	3.3	59.5	1.94	3.98
Op15	669	0.20	0.680	0.744	0.105	0.343	0.950	0.060	0.100	0.979	6661	35.8	47.5	55.3	33.7	3.5	61.5	4.72	38.0	49.5	55.0	38.8	3.3	60.4	1.67	5.59
Op16	658	0.20	0.737	0.640	0.167	0.336	0.804	0.106	0.078	0.980	8000	38.9	53.3	53.8	32.6	3.1	60.9	7.33	39.6	50.8	55.4	38.0	3.3	60.3	2.85	5.58
Op17	664	0.20	0.778	0.713	0.186	0.469	0.947	0.109	0.079	0.860	7921	37.6	53.3	58.1	39	3.0	60.2	4.70	39.6	50.6	55.2	38.9	3.3	59.5	2.23	4.47
Op18	630	0.20	0.685	0.463	0.191	0.421	0.940	0.133	0.062	0.998	7872	41.2	52.9	62	38.3	3.3	60.2	6.13	41.3	55.0	55.5	38.9	3.3	59.2	4.50	2.99
Op19	675	0.20	0.766	0.722	0.142	0.469	0.950	0.083	0.079	0.874	7624	36.7	50.2	53.1	30.2	3.5	61.5	6.67	37.0	49.9	55.3	38.9	3.3	61.0	1.17	6.82
Op20	657	0.20	0.770	0.558	0.186	0.547	0.949	0.134	0.067	0.958	8000	41.3	53.7	60	36.6	3.1	61.4	7.91	40.1	53.7	55.4	39.0	3.3	60.5	3.76	4.17
Op21	649	0.20	0.761	0.520	0.182	0.596	0.935	0.139	0.073	0.961	7951	37.6	52.3	49.4	34.5	3.2	61.1	6.45	40.0	51.6	55.8	38.8	3.4	60.8	3.72	6.65
Op22	648	0.21	0.764	0.536	0.172	0.480	0.950	0.124	0.075	0.997	8000	39.1	53.4	53.6	31.6	3.4	60.8	7.41	39.6	52.5	54.7	38.7	3.3	59.5	3.10	5.43
Op23	649	0.20	0.780	0.619	0.159	0.480	0.919	0.105	0.091	0.966	7222	39.1	51.8	61.5	34.7	3.4	60.1	6.64	39.2	51.5	55.6	37.6	3.4	59.0	3.49	3.44
Op24	668	0.20	0.780	0.847	0.099	0.282	0.950	0.031	0.100	0.859	8000	35.5	47.3	54	33	3.6	61.0	5.80	36.2	45.2	54.9	38.9	3.3	59.3	2.00	6.18
Op25	678	0.20	0.772	0.627	0.155	0.582	0.927	0.120	0.100	0.973	7430	39.9	53	56.1	37	3.4	62.7	5.54	38.4	50.1	55.1	36.1	3.3	60.6	3.04	3.29
Op26	662	0.20	0.763	0.394	0.170	0.597	0.724	0.151	0.044	1.000	8000	41.8	50.1	50.6	33.5	3.6	64.8	9.79	35.6	46.8	50.5	34.3	3.3	59.3	4.48	6.80
Op27	692	0.20	0.780	0.759	0.134	0.700	0.950	0.111	0.100	0.854	8000	36.1	49.8	56.2	28.6	3.3	63.0	5.90	35.6	46.8	52.1	36.5	3.3	59.5	3.05	7.98
Op28	675	0.20	0.731	0.700	0.106	0.592	0.950	0.096	0.100	0.933	8000	34.3	48.2	55.1	31.1	3.6	62.2	6.88	35.6	46.4	52.4	34.8	3.3	59.3	3.83	6.22
Op29	652	0.20	0.503	0.734	0.134	0.200	0.761	0.090	0.067	0.915	8000	38.4	50.1	55.6	31.6	3.5	61.7	6.25	35.7	47.3	50.6	32.9	3.3	59.0	5.01	5.97
Op30	612	0.20	0.762	0.187	0.227	0.216	0.928	0.129	0.035	1.000	7986	29.8	39.7	38.6	32.1	4.8	63.8	22.87	37.0	46.3	51.9	34.3	3.3	59.7	4.93	19.96
Op31	670	0.20	0.694	0.950	0.028	0.144	0.783	0.000	0.100	0.975	8000	31	38	43	28	4.7	63.7	22.78	35.7	46.1	55.4	33.9	3.3	59.5	3.46	20.46
Op32	653	0.20	0.748	0.379	0.176	0.668	0.624	0.164	0.015	0.936	7999	38.4	49.7	51	36.9	3.5	64.2	5.88	35.7	46.4	49.7	31.5	3.3	59.4	6.07	7.34
Op33	678	0.20	0.774	0.950	0.089	0.559	0.925	0.060	0.100	0.826	7904	34.3	46	51.8	28.6	3.8	62.0	10.63	35.3	47.0	55.4	33.4	3.4	59.4	3.94	7.26
Op34	649	0.20	0.780	0.581	0.158	0.670	0.950	0.127	0.060	0.878	8000	37.8	52	53.7	34.1	3.4	60.3	5.09	35.6	47.8	53.4	33.7	3.4	59.3	4.03	2.88
Op35	648	0.20	0.764	0.572	0.140	0.497	0.908	0.104	0.041	0.953	7647	38.7	50.9	51.7	38	3.3	60.5	3.63	35.7	48.3	53.7	31.6	3.3	59.5	4.22	5.87
Op36	657	0.20	0.767	0.631	0.125	0.453	0.946	0.090	0.062	0.962	7896	38.5	49.6	49	26.2	3.6	60.9	10.73	35.5	47.3	52.3	32.7	3.3	59.4	4.47	9.13
Op37	670	0.20	0.729	0.886	0.168	0.339	0.874	0.082	0.078	0.781	7833	37.9	52.7	53.1	38.8	3.4	59.8	3.40	35.6	47.9	54.0	32.0	3.3	59.4	4.01	6.33
Op38	686	0.20	0.756	0.923	0.166	0.473	0.950	0.086	0.100	0.743	7483	39.1	53.1	58.7	30.1	3.3	60.9	7.93	35.5	48.9	53.8	34.4	3.3	59.5	2.79	7.01
Op39	653	0.20	0.469	0.776	0.084	0.491	0.603	0.108	0.049	0.884	7946	34.2	45	49.3	28.2	4.0	63.7	13.42	35.6	47.4	50.5	31.9	3.3	58.7	5.49	8.37
Op40	689	0.20	0.763	0.950	0.033	0.469	0.949	0.033	0.100	0.999	7166	31.5	39.8	45.5	25.7	4.3	65.5	20.66	35.6	47.2	54.8	30.1	3.3	59.4	4.82	16.96
Op41	695	0.20	0.479	0.950	0.110	0.251	0.554	0.066	0.100	0.873	4108	37	47.5	51.6	31	3.7	67.3	10.19	35.6	49.8	53.2	30.0	3.3	59.1	4.56	6.32
Op42	665	0.20	0.780	0.701	0.148	0.426	0.897	0.094	0.084	0.901	7992	37.1	51	57.6	32.2	3.3	60.8	5.03	35.8	47.3	51.6	32.4	3.4	58.9	5.50	4.66
Op43	652	0.20	0.780	0.504	0.161	0.435	0.885	0.113	0.039	0.954	7973	38.5	51.3	55.2	31.5	3.4	61.7	6.24	35.6	47.4	49.8	32.9	3.3	59.2	5.11	6.06
Op44	679	0.20	0.780	0.752	0.165	0.475	0.950	0.089	0.098	0.845	6067	37.03	52.1	58.6	35	3.0	61.4	6.15	36.5	49.9	54.6	32.8	3.3	59.3	3.34	5.36
Op45	695	0.20	0.726	0.878	0.148	0.514	0.715	0.080	0.100	0.797	3420	37.3	51.3	54.4	33.4	3.1	62.7	5.89	35.7	50.6	54.5	31.4	3.3	59.3	3.83	3.95
Op46	674	0.20	0.720	0.883	0.176	0.403	0.818	0.079	0.098	0.724	5174	39.9	52.5	58.7	31.8	3.1	59.7	8.05	35.7	50.0	54.5	33.3	3.3	59.0	2.90	5.80
Op47	683	0.20	0.780	0.702	0.150	0.779	0.717	0.124	0.079	0.821	4826	38.1	49.6	57.9	31.3	3.1	62.8	7.21	36.3	49.7	52.7	32.8	3.3	59.7	3.87	5.01
Op48	671	0.20	0.774	0.643	0.146	0.477	0.950	0.096	0.100	0.979	6233	36.7	50.8	55.9	34	3.2	61.9	4.31	37.0	49.7	54.8	33.1	3.3	59.3	3.37	2.48
Op49	677	0.20	0.780	0.711	0.167	0.552	0.848	0.087	0.100	0.810	3422	39.6	52.2	60.2	32.4	3.2	61.7	8.06	35.5	50.2	54.7	32.9	3.3	59.7	3.05	5.21
Op50	641	0.20	0.768	0.755	0.100	0.632	0.950	0.087	0.025	0.879	7817	35.5	46.1	50.5	34	3.9	59.0	8.00	36.1	49.6	55.4	29.3	3.3	59.6	4.47	8.20
Op51	665	0.20	0.609	0.938	0.118	0.204	0.877	0.039	0.098	0.855	4903	35.4	49	54.3	29.9	3.7	60.1	6.51	35.9	49.7	55.8	30.5	3.3	59.4	3.89	3.06
Op52	636	0.20	0.480	0.730	0.140	0.194	0.950	0.052	0.081	0.926	6581	39.6	51.2	55.7	37.6	3.7	60.2	5.23	39.6	49.5	57.9	38.7	3.3	59.5	2.84	3.71
Op53	653	0.20	0.768	0.568	0.249	0.239	0.950	0.059	0.026	0.670	5688	42.1	53.6	63.6	40.3	2.9	60.2	9.63	36.7	47.7	55.2	38.9	3.3	59.5	1.29	9.26
Op54	648	0.20	0.589	0.540	0.132	0.540	0.950	0.024	0.078	0.829	1	37.3	50.1	55.2	32	3.6	61.0	5.88	36.3	48.6	58.0	38.8	3.6	59.5	2.91	5.95
Op55	655	0.20	0.740	0.728	0.184	0.500	0.950	0.074	0.067	0.671	5834	43.7	54	60.2	32.2	3.2	58.9	10.16	36.8	49.1	56.5	35.5	3.3	59.3	2.66	7.49
Op56	647	0.20	0.658	0.496	0.191	0.481	0.948	0.023	0.088	0.678	6	41	52.5	62.8	38.8	3.3	60.6	6.04	37.5	49.0	57.4	37.7	3.5	59.3	3.51	5.98
Op57	622	0.20	0.658	0.541	0.177	0.299	0.940	0.055	0.028	0.768	5208	40.5	53.6	55.4	36.6	3.4	57.9	5.47	37.1	49.1	55.6	34.4	3.5	59.3	3.96	4.77
Op58	642	0.20	0.780	0.610	0.225	0.100	0.950	0.050	0.040	0.676	5836	42.2	54.8	63.0	37.5	3.0	58.6	9.48	36.1	49.9	55.7	33.6	3.3	59.4	2.86	9.92
Op59	649	0.20	0.644	0.798	0.184	0.100	0.950	0.060	0.064	0.670	7665	40.5	53.3	58.7	42.0	3.1	57.9	7.13	35.6	49.8	55.5	32.4	3.4	60.2	3.38	9.78
Op60	642	0.20	0.776	0.609	0.181	0.349	0.950	0.085	0.072	0.832	7876	37.9	53.7	60.8	36.8	3.2	59.0	5.63	38.4	49.7	55.3	36.8	3.5	59.4	3.09	4.54
Op61	631	0.20	0.769	0.272	0.238	0.100	0.946	0.015	0.038	0.672	1121	39.2	46.4	47.9	39.7	3.9	63.2	9.63	36.0	49.6	56.2	37.4	3.5	59.9	2.49	8.81
Op62	663	0.20	0.780	0.820	0.179	0.100	0.950	0.052	0.064	0.670	8000	40.7	54.6	55.5	34.5	3.1	59.2	6.94	36.6	49.9	55.3	35.7	3.3	59.8	2.11	5.08
Op63	671	0.20	0.780	0.950	0.155	0.243	0.950	0.054	0.100	0.670	7998	38.4	52.3	61.6	42.2	3.1	59.5	6.52	36.7	49.8	55.4	33.6	3.3	59.4	3.01	8.00
Op64	667	0.20	0.780	0.787	0.150	0.749	0.949	0.096	0.087	0.670	6280	41.4	52.2	51.3	32.4	3.5	60.1	8.90	37.4	49.8	56.5	34.8	3.3	59.5	2.97	6.59
Op65	622	0.20	0.758	0.500	0.220	0.100	0.950	0.056	0.004	0.670	8000	42.9	54.5	55.8	38.6	3.3	58.1	5.81	39.3	49.9	55.3	38.6	3.4	59.5	2.44	3.64
Op66	664	0.20	0.780	0.950	0.136	0.242	0.950	0.051	0.100	0.670	8000	38.1	50.0	56.3	25.0	3.3	59.3	7.67	35.6	50.0	57.1	38.0	3.3	59.2	1.25	10.41

Op67	622	0.21	0.588	0.478	0.156	0.211	0.909	0.088	0.048	0.915	7189	41.1	52.4	53.8	33.9	3.5	59.5	6.92	36.5	51.3	54.3	40.0	3.4	60.3	2.30	6.14
Op68	642	0.21	0.546	0.505	0.146	0.463	0.813	0.105	0.065	0.816	5183	39.2	50.2	51.3	30.4	3.5	61.3	8.19	35.1	45.0	54.8	38.8	3.1	60.8	3.39	11.13
Op69	640	0.20	0.727	0.711	0.132	0.464	0.950	0.099	0.096	0.998	7583	38.2	50.9	58.0	30.1	3.5	58.7	7.20	35.4	49.1	52.3	36.4	3.3	58.1	2.96	8.19
Op70	612	0.20	0.597	0.400	0.247	0.201	0.802	0.140	0.028	0.870	7997	44.8	52.4	57.3	41.2	3.5	59.8	7.84	35.6	50.9	55.0	38.7	3.4	62.8	1.96	6.96
Op71	679	0.20	0.746	0.747	0.121	0.361	0.919	0.045	0.099	0.805	6820	35.9	49.5	53.1	33.7	3.4	61.9	4.41	34.7	50.4	55.4	38.0	3.2	61.7	2.01	4.64
Op72	706	0.20	0.747	0.902	0.151	0.464	0.875	0.062	0.089	0.823	6055	39.5	51.4	53.7	38.6	3.2	63.2	4.47	35.4	49.8	55.5	38.8	3.3	63.4	1.28	3.34
Op73	642	0.20	0.577	0.768	0.198	0.446	0.724	0.136	0.040	0.874	6778	42.4	53.9	56.9	39.7	3.1	59.3	6.41	35.5	49.8	55.6	38.9	3.3	60.3	0.44	6.08
Op74	656	0.20	0.684	0.565	0.212	0.203	0.871	0.112	0.059	0.890	7860	41.1	54.2	59.2	34.4	3.2	61.2	7.92	35.7	49.8	55.4	38.9	3.3	61.0	0.50	7.15
Op75	660	0.20	0.774	0.703	0.143	0.664	0.937	0.126	0.100	1.000	7913	36.5	51.2	57.7	30.4	3.5	60.6	6.31	35.6	50.0	55.4	38.9	3.3	60.8	0.46	6.92
Op76	649	0.20	0.567	0.524	0.170	0.313	0.816	0.110	0.063	1.000	6074	40.3	51.9	50.5	34.9	3.4	62.3	7.29	35.6	49.3	55.4	38.8	3.3	61.7	0.87	6.82
Op77	635	0.20	0.775	0.486	0.197	0.320	0.940	0.103	0.065	0.840	7996	41.4	53.0	57.5	37.8	3.3	59.9	5.28	35.6	51.2	55.4	38.9	3.3	60.1	0.66	4.29
Op78	649	0.20	0.354	0.768	0.143	0.144	0.940	0.099	0.090	0.812	3122	38.6	48.1	48.1	38.8	4.0	63.9	9.06	35.6	49.8	55.2	38.9	3.3	59.4	0.08	8.51
Op79	654	0.20	0.625	0.577	0.175	0.179	0.866	0.095	0.066	0.912	8000	39.7	53.3	54.5	39.6	3.3	61.2	4.37	36.2	49.8	55.4	38.9	3.3	61.0	0.75	3.38
Op80	650	0.21	0.718	0.484	0.166	0.312	0.797	0.070	0.081	0.868	6071	39.1	51.9	53.6	33.0	3.4	61.7	6.47	35.7	50.1	55.6	38.9	3.3	59.5	0.22	6.67
Op81	648	0.20	0.688	0.707	0.145	0.375	0.793	0.100	0.051	0.996	7025	39.3	52.7	53.0	32.4	3.4	59.7	6.75	35.5	50.1	55.4	38.9	3.3	60.0	0.35	7.08
Op82	654	0.20	0.696	0.584	0.157	0.379	0.923	0.095	0.068	1.000	5251	38.2	52.4	59.8	35.5	3.2	61.1	5.78	35.7	50.8	55.4	38.9	3.3	60.4	0.66	5.08
Op83	643	0.20	0.760	0.525	0.242	0.134	0.929	0.100	0.052	0.843	7121	43.9	54.4	62.9	45.4	3.0	60.2	12.18	35.5	49.9	55.4	38.9	3.3	60.3	0.50	10.40
Op84	622	0.20	0.720	0.469	0.184	0.300	0.950	0.102	0.076	0.977	7313	41.1	53.5	53.9	36.4	3.4	59.2	5.93	35.6	50.1	55.4	38.9	3.3	59.2	0.23	5.51
Op85	664	0.20	0.662	0.526	0.149	0.463	0.950	0.107	0.081	1.000	5750	35.0	51.0	56.9	37.0	3.3	62.7	2.98	35.6	50.7	55.4	38.9	3.3	62.7	1.23	1.80
